# A Protocol for a Comprehensive Monitoring and Evaluation Framework With a Compendium of Tools to Assess Quality of Project ECHO (Extension for Community Healthcare Outcomes) Implementation Using Mixed Methods, Developmental Evaluation Design

**DOI:** 10.3389/fpubh.2021.714081

**Published:** 2021-09-21

**Authors:** Smita Ghosh, Brenna M. Roth, Irene Massawe, Emmanuel Mtete, Jacob Lusekelo, Eve Pinsker, Steven Seweryn, Patrick K. Moonan, Bruce B. Struminger

**Affiliations:** ^1^Division of Global HIV and Tuberculosis, US Centers for Disease Control and Prevention, Atlanta, GA, United States; ^2^School of Public Health, University of Illinois, Chicago, IL, United States; ^3^Center for International Health, Education, and Biosecurity, University of Maryland School of Medicine, Baltimore, MD, United States; ^4^Division of Global HIV and Tuberculosis, US Centers for Disease Control and Prevention, Dar es Salaam, Tanzania; ^5^Ministry of Health, Community Development, Gender, Elderly, and Children, Dodoma, Tanzania; ^6^School of Medicine, University of New Mexico, Albuquerque, NM, United States

**Keywords:** evaluation framework, implementation, quality improvement, program monitoring, mixed methods, developmental evaluation

## Abstract

**Introduction:** The United States Centers for Disease Control and Prevention (CDC), through U.S. President's Emergency Plan for AIDS Relief (PEPFAR), supports a third of all people receiving HIV care globally. CDC works with local partners to improve methods to find, treat, and prevent HIV and tuberculosis. However, a shortage of trained medical professionals has impeded efforts to control the HIV epidemic in Sub-Saharan Africa and Asia. The Project Extension for Community Healthcare Outcomes (ECHO^TM^) model expands capacity to manage complex diseases, share knowledge, disseminate best practices, and build communities of practice. This manuscript describes a practical protocol for an evaluation framework and toolkit to assess ECHO implementation.

**Methods and Analysis:** This mixed methods, developmental evaluation design uses an appreciative inquiry approach, and includes a survey, focus group discussion, semi-structured key informant interviews, and readiness assessments. In addition, ECHO session content will be objectively reviewed for accuracy, content validity, delivery, appropriateness, and consistency with current guidelines. Finally, we offer a mechanism to triangulate data sources to assess acceptability and feasibility of the evaluation framework and compendium of monitoring and evaluation tools.

**Expected impact of the study on public health:** This protocol offers a unique approach to engage diverse group of stakeholders using an appreciative inquiry process to co-create a comprehensive evaluation framework and a compendium of assessment tools. This evaluation framework utilizes mixed methods (quantitative and qualitative data collection tools), was pilot tested in Tanzania, and has the potential for contextualized use in other countries who plan to evaluate their Project ECHO implementation.

## Background

The United States Centers for Disease Control and Prevention (CDC) partners with more than 50 countries to improve methods to find, treat, and prevent HIV and tuberculosis (TB) (1, 2). However, a shortage of trained medical professionals remains a major barrier in controlling the HIV epidemic in low- and middle-income countries (3–6). Gaps also remain in training, staff capacity, service delivery, and managing complex disease conditions among people living with HIV (PLHIV) (5–8). Clinical specialists practice primarily in large referral hospitals and rarely extend services into remote areas that are mostly served by small peripheral health units. In addition, specialists are not able to provide regular mentorship and supervision of health care workers in remote areas. Innovative approaches are needed to fill gaps in human resource (8). The Extension for Community Healthcare Outcomes (ECHO^TM^) Project aims to address these gaps through a collaborative hub and spoke model to connect a multi-disciplinary team of health professionals and enable virtual communities of practice (9).

ECHO creates a global network of health workers at local, regional, and international medical centers to help disseminate best practices and improve health outcomes (9). The ECHO model has four components: (1) multipoint videoconferences to leverage healthcare resources; (2) outcome-focused disease management; (3) share best practices, case-based learning to encourage collaborative patient management between local practitioners, their peers, and subject matter experts (SMEs); and (4) systematic methods to monitor outcomes (10). ECHO offers an opportunity for local clinicians in remote healthcare settings to seek guidance and support from national and international SMEs. CDC's Division of Global HIV and TB supports utilization and expansion of the ECHO model for mentoring, virtual technical assistance, and knowledge dissemination to strengthen effective HIV and TB service delivery. CDC is supporting implementation of this approach in more than 23 countries across six continents (9).

The importance of maintaining high-quality implementation was studied for education, technology, manufacturing, and service industry (3, 4, 11). Quality of healthcare implementation is affected by various factors related to patients, providers, communities, and the type of intervention or innovations (11–13). ECHO program processes, outcomes, and continuous quality improvement related to delivery, organizational functioning, trainings, technical assistance, and mentoring, are all essential features of program implementation and evaluation (11). High-quality implementation should be the standard for program replication and expansion (14). It is necessary to understand elements that support high-quality ECHO implementation and develop a framework to monitor progress, assess performance, and develop explicit recommendations for broader scale-up, replication, and sustainability. This manuscript describes a practical protocol, evaluation framework, and compendium of tools to assess implementation of Project ECHO.

The proposed evaluation framework and compendium of tools will be pilot tested in Tanzania. Tanzania is one of 13 PEPFAR high-priority countries with 1.6 million PLHIV and an estimated 5% prevalence of HIV in the adult general population (1). As of August 2019, an estimated 78% of PLHIV in Tanzania knew their HIV status (15). Among these, 92% were on treatment (~71% of all PLHIV) and, 87% were virally suppressed (~62% of all PLHIV) (8). Despite the efforts to meet PEPFAR targets, structural, legal, and social barriers to HIV services exist (16). According to 2016 estimates provided by the World Health Organization (WHO), Tanzania had one of the lowest physician-to-patient ratios in the world, with 0.14 medical doctors per 10,000 people (8). The lack of doctors and medical professionals was more pronounced in rural areas, where there were often no medical professionals available to manage complex HIV patients with cardiovascular diseases, diabetes, TB, and drug-resistant TB (5, 6).

Tanzania has implemented an HIV ECHO clinic in an ‘all teach, all learn' interactive format. The University of Maryland, Baltimore (UMB) has facilitated more than 76 weekly ECHO sessions between November 2018 through July 2020 across the country.

## Evaluation Design and Objective

**Figure 1:**
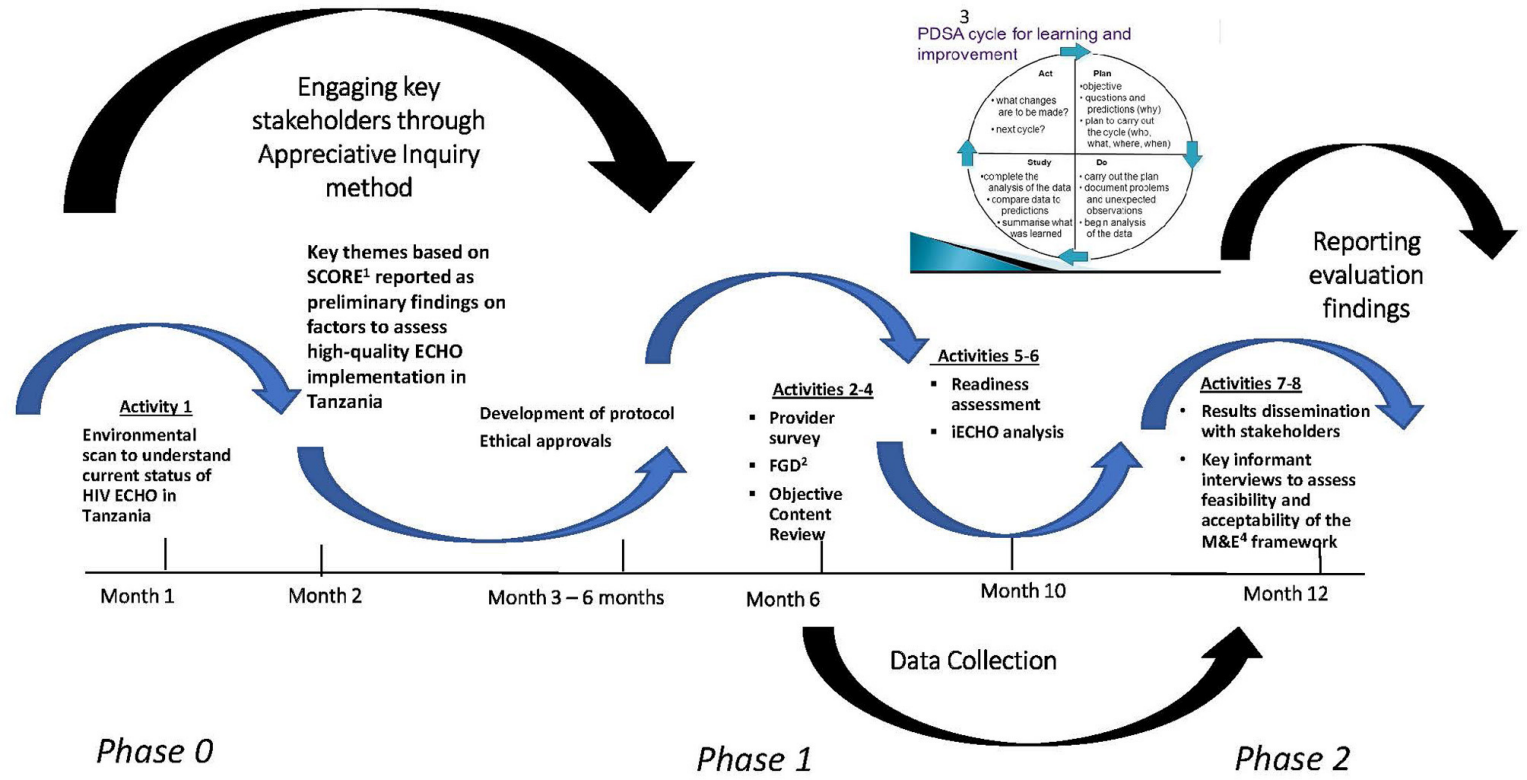
Evaluation implementation phases. ^1^SCORE, Strengths, Challenges, Opportunities/Aspirations, Measurable Results, Evaluation; ^2^FGD, Focus group discussions; ^3^PDSA, Plan, Do, Study, Act; ^4^M&E, Monitoring and evaluation.

**Figure 2:**
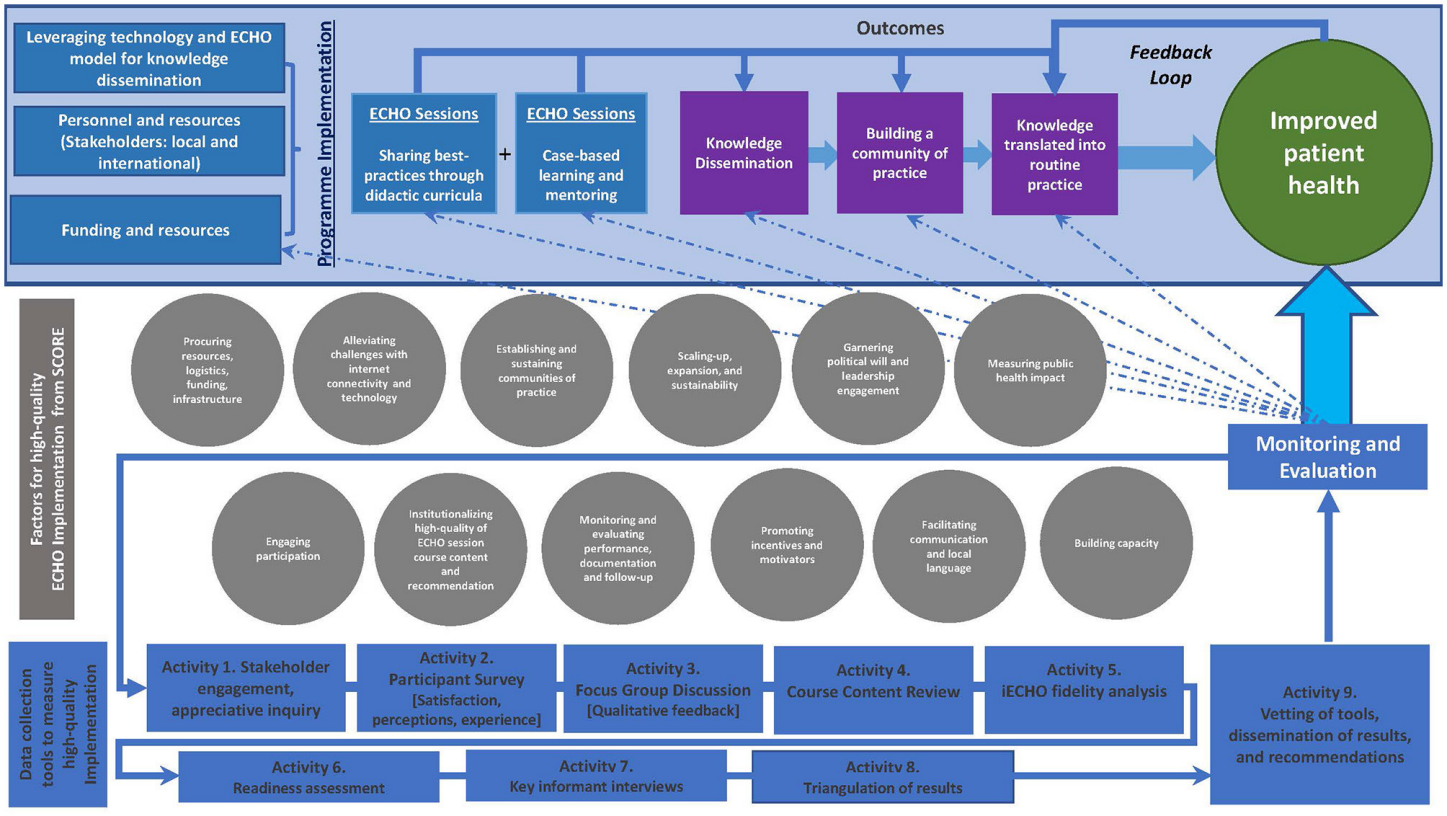
Proposed Comprehensive Evaluation Framework with evaluation activities.

This is a multi-phased, mixed-methods (17, 18), developmental evaluation design (17, 19, 20) (Figure 1). A pragmatic formative process evaluation using appreciative inquiry approach (21, 22) will assess stakeholder perceptions of participation, engagement, satisfaction, learning, self-confidence and applying knowledge acquired in ECHO sessions to practice. Additionally, review of the ECHO sessions will assess quality of facilitation, content, interactivity during sessions, and applicability of recommendations provided. This comprehensive evaluation framework and compendium of tools will assess quality of an HIV ECHO implementation in routine practice that may be adapted for evaluation of any ECHO program. Specific evaluation questions are presented in Box 1. Figure 2 and Appendices 1–9 includes data collection tools.

Box 1 **Evaluation questions.**

1.) What are the current stakeholder perceptions of high-quality HIV ECHO implementation?
  1.1.) How do HIV ECHO participants and implementers (Health care providers, faculty, ECHO organizers) perceive high-quality implementation?
  1.2.) How do HIV ECHO participants and implementers perceive their level of engagement in the HIV ECHO program?
  1.3.) How do HIV ECHO participants and implementers perceive their level of satisfaction with HIV ECHO program?
  1.4.) How do HIV ECHO participants and implementers perceive their learning?
  1.5.) How do HIV ECHO participants and implementers perceive their self-confidence in managing complex HIV patients?
  1.6.) How do HIV ECHO participants and implementers perceive their level of competence?
  1.7.) What do HIV ECHO participants and implementers perceive are potential barriers to HIV ECHO participation?
  1.8.) To what degree has HIV ECHO sessions influenced behavior of the participants?
  1.9.) Was the content of previous HIV ECHO didactic presentations, accurate, clear, and valid?
  1.10.) Was the content of previous HIV ECHO case-based presentations, accurate, clear, and valid?
  1.11.) Were case-based recommendations made by the expert panel applicable to the case presented, appropriate, useful, and, relevant?
2.) To what extent does the draft evaluation framework and compendium of tools describe, measure, and validate the described elements (constructs) to effectively monitor a high-quality HIV ECHO implementation routinely?
3.) What are the recommendations for improving the proposed evaluation framework and the compendium of monitoring and evaluation tools?

## Evaluation Activities

### Phase 0

#### Activity 1: Environmental Scan, Stakeholder Engagement, and Tool Development

We will engage all stakeholders using a participatory approach using a modified appreciative inquiry methodology (18) that will guide an open discussion that elicits a variety of perspectives (strengths, challenges, opportunities, long-term aspirations, and measurable results, evaluation: SCORE) from key stakeholders. Details of this appreciative inquiry approach is presented elsewhere (23).

### Phase 1

#### Activity 2: Participant Survey

We will use an anonymous, on-line, standardized survey to better understand the perceptions and experiences of participants of the HIV ECHO program (Appendix 1). Questions are designed to gauge participant engagement, level of satisfaction with the HIV ECHO program delivery, perceived learning, perceived self-confidence in managing complex HIV patients, perceived competence, potential barriers to HIV ECHO participation, and the degree to which the HIV ECHO clinic has influenced participants to translate the knowledge they gained into practice (Appendix 1). Upon consent (Appendix 1), we will deploy the survey using the Qualtrics® (Qualtrics, Provo, UT, www.qualtrics.com) platform. Email addresses will be obtained from participant registration forms and the iECHO database. All ECHO participants will be sent an email containing a link the web-based consent form and survey questionnaire. The survey will take approximately 30 min to complete.

#### Activity 3: Focus Group Discussions

We plan to conduct an exploratory cross-case analysis between the themes that arise among the three different groups (ECHO session attendees, subject matter experts who present at ECHO sessions, and ECHO administrators who are health care providers may have different perspectives on ECHO). Three focus group discussions (FGDs) will be conducted with each group comprising of ECHO participants who would have attended ECHO in at least the past year. Each FGD will consist of five to six participants. Each of the three groups will be comprised of participants who have similar backgrounds, job roles and experience to ensure that all participants are comfortable with participating and openly contribute to facilitated discussions (Appendix 2A,B). Guided, well-structured discussions will probe into findings from the survey, to describe how they may be translating knowledge into practice (Appendix 2A–C).

Participant names will not be recorded or retained to ensure confidentiality. The focus group facilitator will explain the evaluation objectives, procedures, risks, benefits, and the informed consent process. Individuals will be provided an opportunity to ask questions before consenting (Appendix 3). The focus groups will be conducted in-person in Kiswahili and/or English, depending on language skills of the facilitator and participants. Participants will be informed that the session's audio will be recorded digitally. Recorded interview sessions will be stored on a secure cloud-based repository. A focus-group observer will assist the facilitator with recording and note-taking. To mitigate the possibility of FGD participant identity being disclosed and potentially resulting in stigma for participants, discussions will be held in a private setting with only evaluation participants in audience. At the beginning of each focus group, facilitators will reiterate the intended goals of the focus group. The facilitator will clarify that, as the project is an operational evaluation, there are no “right” or “wrong” perceptions or thoughts with regards to feasibility. The facilitator will also ask participants to extend professional courtesy to their fellow participants and maintain the confidentiality of the conversation or thoughts expressed in the FGDs by not sharing them with others.

#### Activity 4: Objective Review of Session Content

A peer-reviewed concept shall be utilized to validate the educational content, improve standards of quality, and provide credibility in a transparent review process. Select HIV experts from low- and high-HIV prevalence countries will be invited to participate to maintain an impartial, fair, and objective assessment. Eligibility criteria for objective reviewers will include being familiar with the model, philosophy, and functioning of Project ECHO; Being a physician, nurse, or health care provider who knows the clinical guidelines of the relevant country and programmatic operations for HIV, TB, PEPFAR, or other relevant programming; Being fluent in the local language (e.g., Kiswahili for ECHO clinic in Tanzania) in which ECHO sessions are conducted; Not being an employee or volunteer for the organization that implements the Project ECHO clinic(s) being evaluated; Not being affiliated with the organization that funds or competes with funding for the Project ECHO clinic(s) being evaluated; Not being a participant or subject matter expert (SME) of the Project ECHO clinic(s) being evaluated. The objective reviewer can, however, be a SME for a Project ECHO clinic not being evaluated.

Each reviewer will assess three sessions each, and each session will have three reviewers. See Table 1 for matrix of reviewer and session selections.

**Table 1:** Matrix for objective reviewer and ECHO session selection.

	**Reviewer 1**	**Reviewer 2**	**Reviewer 3**	**Reviewer 4**	**Reviewer 5**	**Reviewer 6**
Session 1	X				X	X
Session 2	X	X			X	
Session 3	X	X	X			
Session 4		X	X	X		
Session 5			X	X		X
Session 6				X	X	X

##### Facilitator Scorecard

Session recordings shall be randomly selected from previously conducted HIV ECHO session recordings for an objective review of the facilitation process using a standardized scorecard (Appendix 4A). An independent panel of reviewers will use the scorecard to objectively rate the facilitator on coordination of the session following the ECHO guidelines, engagement of participants, insights offered, and time management. Each reviewer will be given written instructions and a scorecard to rate the facilitator for their assigned session.

##### Didactic Presentation Review

A random selection of previous ECHO session recordings shall undergo an objective review for didactic presentation accuracy, content validity, and delivery (Appendix 4B). An independent panel of reviewers (both national and international experts) will be given written instructions and a scorecard to rate the didactic presentation for their assigned session. Each selected session will be reviewed using a standardized tool to assess the following: extent to which the session achieves stated learning objectives; accuracy and validity of didactic presentations compared to recommended clinical practices as outlined in the most up-to-date national and international guidelines; presentation quality (e.g., free from errors, effective, and engaging communication).

##### Case-Based Presentation and Recommendation Review

An independent panel of reviewers will use a standard scorecard to assess case presentations from a random selection of ECHO sessions. Each selected session will be reviewed for the following: (1) extent to which the case-based presentation aligned with stated learning objectives; (2) presentation quality (e.g., free from errors, effective and engaging communication); (3) appropriateness of the recommendations; (4) whether the recommendations were specific, measurable, achievable, reproducible, and time bound; (5) whether the recommendations were consistent with current national and international guidelines for standard practices of care (Appendix 4C). At the conclusion of the individual scoring and assessments, the reviewers shall be convened to discuss convergence and divergence and give in-depth feedback on their review process and content.

#### Activity 5: Review of Routinely Collected Programmatic Data Including iECHO Analysis

Secondary de-identified data (e.g., number of participants, number of partners represented, timing of sessions, notable technical challenges) will be analyzed to identify trends and monitor performance over time (Appendix 5). Appropriate use, identification of use by stakeholders, frequency of data sharing between stakeholders, and overall utility of iECHO will be assessed and discussed.

#### Activity 6: Readiness Assessment

A trained evaluation team member from the project team will administer readiness assessment questionnaires of newly enrolled ECHO site coordinators through face-to-face interviews with ECHO program coordinators preparing to launch new ECHO hubs (Appendix 6).

### Phase 2

#### Activity 7: Key-Informant Interviews

Semi-structured interviews will be conducted with key-informants to obtain personal insights based on their experience and perspective (Appendix 7). Key informants shall be consented, given the framework, tools, and results from data collection in advance of the key informant interview (KII) (Appendix 8). They will be asked to provide written feedback to assess acceptability, feasibility, validity, and reliability of the compendium of tools and whether these tools capture the elements of high-quality ECHO implementation (Box 2). During the KII, participants will be probed by the Principal Investigator or the co-Principal Investigator to expand on their written responses and offer points of clarification and suggestions for improvement.

Box 2 **Key informant questions.**

These interviews will assess the (i) utility (*Who needs what information?*), (ii) feasibility (*How much money, time, effort is needed to be conducting this evaluation routinely?*), (iii) propriety (*What steps can be taken for evaluation to be ethically conducted with regard to those involved and those affected?*), (iv) accuracy (*What design will lead to accurate information being collected?*).

#### Activity 8: Triangulation

**Figure 3:**
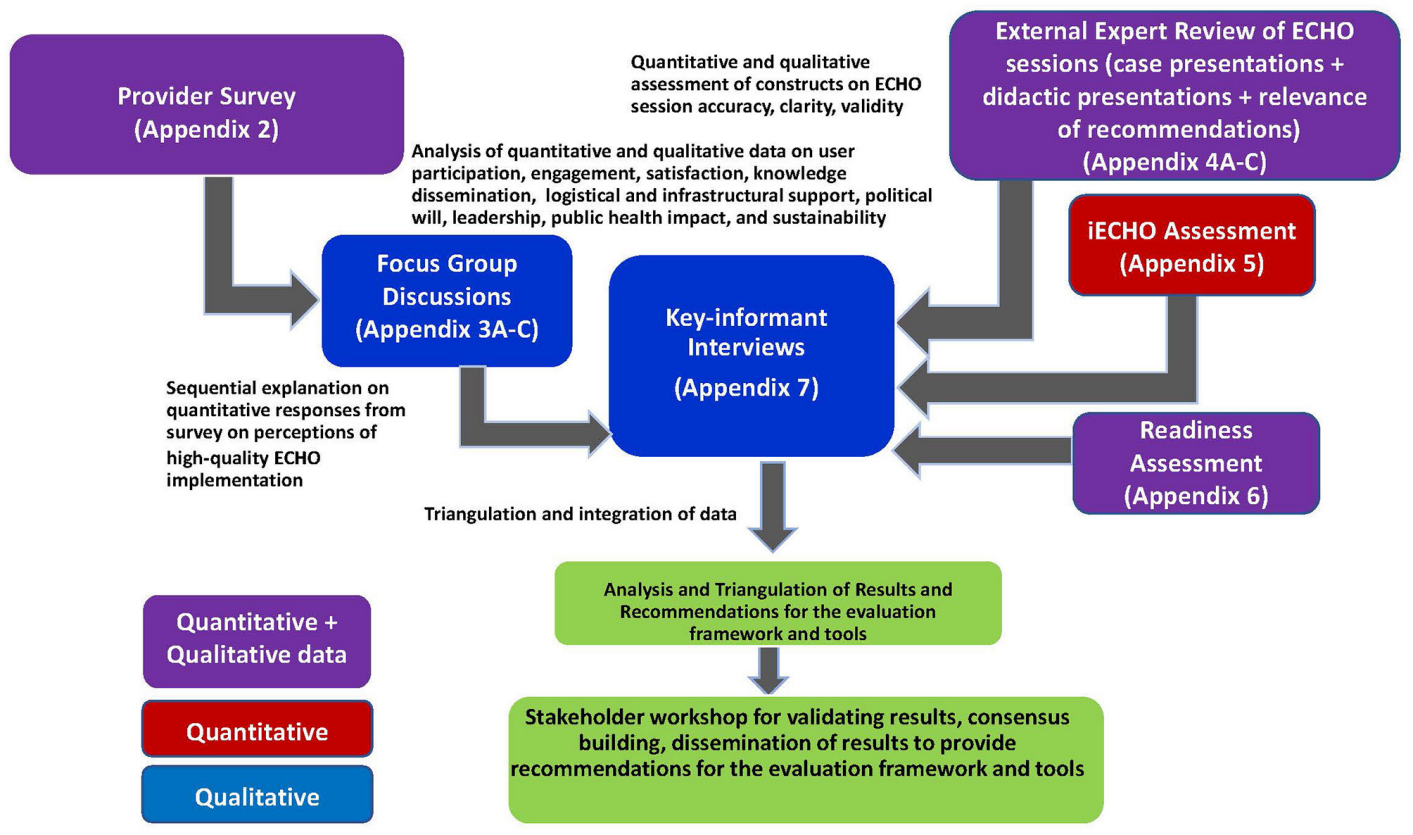
Triangulation of the key concepts from qualitative and quantitative data collection tools.

Evaluation investigators will triangulate the data collected during Activities 2–7 and revise the framework and tools accordingly (Figure 3). Convergence and divergence of themes stratified by implementers shall help finalize the findings. Triangulation will ascertain patterns of convergence, or divergence of findings through different data sources as well as help in understanding of inconsistencies, thus providing opportunities for deeper insight into the relationship between the quantitative and qualitative data collected (24, 25). A final practical manual of the framework, compendium of tools, when and how to use them, and lessons learned from this evaluation shall be the final deliverable.

#### Activity 9: Stakeholders Meeting to Discuss Results, and Development of a Dissemination Plan

A final stakeholder workshop of ~30 participants, including those present for Activity 1 (Stakeholder Workshop) shall be invited to discuss the results from all the activities above. This interactive workshop-style meeting shall be organized at the completion of data analysis and triangulation to present evaluation findings. Facilitated discussion of feedback on usability and acceptability of the tools and framework adoption routinely shall be sought by key stakeholders, some of whom would be the same participants who attended the environmental scan workshop. Tools, POC, and frequency of data collection shall be recommended as the final deliverable.

## Inclusion Criteria

Online survey: All participants who have attended at least one Tanzania HIV ECHO session facilitated by UMB are eligible to participate.

Focus group discussion: All participants who have attended at least one HIV ECHO session and completed the survey are eligible to participate. Twenty healthcare providers (HCP) and SMEs and/or implementers shall be randomly selected from the iECHO database and invited to participate in the FGD. Five to six participants shall be randomly selected from those who agree to participate. There will be three FGDs (one each with SMEs, one each with HCPs, one each with implementers), each comprising of 5–6 people in each group. Implementers could be ECHO coordinators who are primarily responsible for coordinating and delivering the ECHO sessions routinely. Location of the interviews shall be pre-determined, and invitations shall be sent 1 month in advance.

Readiness assessment: Two coordinators of new HIV ECHO hubs shall complete the readiness assessment checklist. These coordinators will be selected from those who have not started implementing their HIV ECHO hubs.

Key-informant interviews: six purposively chosen opinion leaders or influencers (e.g., administrator from MOH, or UMB, funder, manager of a health clinic that implements ECHO, private provider who was a SME for one of the ECHO sessions) who can advocate for ECHO from public, private, academic, and Ministry of health (MOH) shall be invited for interview.

## Exclusion Criteria

Persons who have never attended a Tanzania HIV ECHO session shall be excluded from participation in any data collection efforts.

### Recruitment and Enrollment Procedures

Once the evaluation has been approved, the evaluation co-investigators will announce the opportunity to participate during an upcoming HIV ECHO session. Participant emails will be furnished by UMB, who routinely collect contact information at the start of ECHO sessions. Stakeholders who attended the stakeholder workshop (Activity 1) shall advocate and encourage participation in the online survey for all ECHO participants by announcements and reminders at monthly ECHO sessions. Gentle reminder emails once at the end of 1 month, and again 1 week before the 2-month deadline shall be sent to increase response rates for the online survey (Activity 1). All other activities shall include random selection of participants, or a purposeful invitation of expert faculty, ECHO participants, and implementers from the iECHO database.

For the FGD (Activity 3), 5–6 participants will be selected for each group from among participants with similar roles and responsibilities; they will be selected only if they completed the above ECHO participant survey.

For the key informant interviews (Activity 8), six key stakeholders (opinion leaders) will be selected amongst those engaged in ECHO implementation: two representatives from the two ECHO participant groups of HCP and SME, a representative from UMB and one from CDC-TZ, one from Tanzanian MOH and one UMB administrator be invited to review summaries of results of Activities 1–7, and all data collection tools.

### Sample Size

Since data collected is not intended to be generalizable, no formal sampling frame is required. Purposeful sampling (26) will be used for most activities, except for Activity 2 (Participant Survey). The participant survey will be sent to all persons that attended at least one HIV ECHO session during the evaluation period. We estimate 300 participants will be eligible, with at least 75% response rate, resulting in an anticipated sample size of 225 respondents (Table 2). For all other qualitative activities, purposeful sampling approach shall be undertaken.

**Table 2:** Sample size table with activities and inclusion criteria.

**Activity number and description**	**Inclusion criteria**	**Expected number of participants**	**Rationale for the number of participants**	**Selection method**
1—Environmental scan workshop	Convenient sample of invitees, representatives from hub and spoke sites	40	Key representatives from groups of interest, i.e., Implementers (Administrators, Policy makers), Subject matter experts (Presenters, facilitators), ECHO participants who are Health Care Providers, a 6-month check in what was working and what weren't.	Convenient representative sample from hubs and spokes
2—Online survey	All participants attending at least one ECHO session.	225	All (census)	All (census)
3—Focus group discussions	15–18 participants: 5–6 participants selected randomly for 3 FGD among those who answered the online survey: (i) health care providers; (ii) community health workers and laboratorians; (iii) presenters of didactic sessions or case studies	Three focus group discussions (one each for implementer, physicians subject matter experts, and healthcare providers) with 5–6 participants per FGD for a total of 15–18	Three FGDs needed to cover each of one each for healthcare providers, community health workers/laboratories, and facilitators/presenters; 5–6 per FGD felt to foster open discussion but be manageable, based on the experience of the investigators.	Stratified random sampling from the iECHO database by category
4—Objective review of sessions	Purposeful sample of 6 reviewers (Tanzanian and international HIV experts and communication specialists) who would review 3 sessions each.	3 sessions selected randomly reviewed by 6 reviewers	Since the experts are very busy, and volunteering their time, having them review 3 sessions and then spending 1 h for introduction and 1 h for debriefing after the results are shared by them seemed a reasonable expectation for their time commitment.	Purposeful sampling
5—Routinely collected programmatic data review	100% inclusion of all iECHO records	All sessions covered from Nov 2018 to December 2019	Reviewing all data will be important to understand the potential and propose effective use of this data that is being collected.	All data included in analysis
6—Readiness assessment	ECHO coordinators from 2 new programs	2	Conducting this interview shall provide a sense of questions that are relevant for readiness assessment	Convenient relevant sample on eligible ECHO coordinator
7—Key-informant interviews	Purposeful sampling of 6 key decision makers	6	Representatives from implementers (2) Ministry of Health (2), University of Maryland, Baltimore (1), health care providers (1) and subject matter experts (1) shall review all results and tools and help define high-quality implementation and feasibility of the proposed framework	Purposeful sampling
8—Triangulation	Data shall be triangulated and consensus reached between 6 investigators		All (census)	All (census)
9—Final stakeholder workshop	All “key” stakeholders including some who attended the environmental scan workshop	~30	A representative group of hub and spoke participants (hopefully the same participants from the stakeholder workshop where we began development of this evaluation framework and tools). This would help close the loop on results dissemination and providing evidence for the evaluation framework and tools.	Purposefully Selected

*FGD, focus group discussions*.

### Data Analysis

Descriptive statistics will be used to describe and summarize participant characteristics, perceptions, and self-efficacy measures of the HIV ECHO clinic. Chi-square statistics shall be used to assess potential relationships between select characteristics of the participants and outcomes of HIV ECHO based on satisfaction and knowledge gained. Descriptive statistics for ordinal ratings (e.g., Likert scales) for Activities 2, 4, and 6 shall be generated using simple frequencies or percentages, and medians or modes will be used as the measure of central tendency. Non-parametric statistical techniques will be employed (e.g., Kruskal-Wallis). Based on the results, the 4-point scales may be recoded for simplicity of reporting. Stratified analysis between different groups (implementers and participants) will clarify relationships of the variety of perceptions on efficacy, satisfaction, and knowledge gain.

Results from the quantitative data shall be explored further through the FGDs. A systematic qualitative analysis will summarize individual and group reflections. Content analysis will include searching for *a priori* codes and recurring themes. Coding and thematic analysis shall be conducted for the qualitative data and compared and contrasted with the survey data. Auto coding using MAXQDA (VERBI, GmbH; Berlin, Germany) will help with thematic analysis. Contextual or cross-case analysis for the significant codes shall be conducted between the various stakeholder roles. Understanding the alignment or discordance of the *a priori* codes among the different stakeholders will be key. Data-reduction efforts shall be undertaken to eliminate ancillary information that did not appear to be significant or relevant. Emergent codes identified will lead to revising the original code book (21, 26, 27). Additionally, there will be voice recordings of interviews and meetings, FGDs, object review debriefings, and readiness assessment interviews that shall be transcribed professionally and analyzed by at least two investigators to ensure inter-coder reliability of >90% generated by MaxQDA kappa statistics.

Triangulation—broadly defined as the comparison of results from multiple data collection methods and/or sources in the evaluation of the same phenomenon—shall be used to document and understand convergence and divergence of findings between the different data collection tools (28). Various concepts/variables/findings shall be further examined and stratified to understand multi-level variations in perceptions and performance. Triangulation shall be used for cross-checking for internal consistency or reliability, as well as “*between-method*” triangulation to test the degree of external validity (24, 25). Data comparison will then help explain convergence and divergence of results between and among different data collection methods and constructs.

For this qualitative data analysis, steps of data reduction, transformation, comparison, and integration will be followed. Data from two different sources (survey and FGD) is stronger than one data source alone, further probing of main concepts of interest from survey during FGD, will help better understand the results (24). Since the previous results will be examined across the KIIs, the final presentation of results to the larger group of stakeholders shall inform development of final recommendations for high-quality ECHO implementation.

### Data Ownership, Security, Storage, and Retention

All evaluation and programmatic data will be owned by the Tanzania MOH or the implementing partner (UMB). No individual, personally identifiable information shall be collected, analyzed, or reported. All data will be kept in password protected computers, only accessible to co-investigators. Audio recordings of focus group discussions (Activity 3) shall be destroyed after data analysis and translation. Audio recordings and paper-based data will be stored under lock and key in cabinets with access limited to authorized evaluation personnel (UMB evaluation team and limited CDC project officer or principal investigators). Management of data shall be restricted to key personnel. All project information will be kept confidential and will be available only to authorized users involved in the evaluation project. Each evaluation personnel will sign a confidentiality agreement indicating that he/she has been instructed in confidentiality procedures under their MOH public health program jurisdiction/implementing partner protocols and will observe them (Appendix 9). Aggregated data and anonymous quotations shall be reported or published for results of evaluation. A copy of all de-identified data shall be retained at local CDC office until all analyses have been completed and planned manuscripts are published. Then all data shall be archived under the MOH policies as custodian of the data. The evaluation framework and compendium of tools will be made publicly available through the University of New Mexico Health Science Center (Project ECHO) website or cloud-based file sharing platforms.

### Ethics and Protection of Human Subjects

Ethical approvals may require local and institutional approvals prior to implementation. This protocol was reviewed by National Institute of Medical Research in Dar es Salaam, Tanzania; the University of Illinois in Chicago, United States; and the University of Maryland, Baltimore, United States; and the Human Subjects Office within the Center for Global Health at the United States Centers for Disease Control and Prevention (CDC). For adoption in other countries or PEPFAR-funded programs, a protocol amendment may be sought from CDC, Atlanta to document any locally required changes.

Project staff will obtain informed consent (Flesh-Kincaid Reading Level for Consent 7.5) for those individuals willing to participate in the evaluation activities. Consent forms will be in English and the local language, Kiswahili, describing the evaluation details, procedures, risks, and benefits. Forms will be translated from English to Kiswahili, then back to English to verify that nothing was lost or altered in a substantive manner during translation. The individuals will be asked to read and review the document. If the participant is not able to read the document or has low literacy skills, the consent form will be read aloud by the evaluation staff and individuals will be offered an opportunity to ask questions about the evaluation and the consent process.

It will be emphasized that evaluation participation is voluntary and that either agreeing or declining to participate in the evaluation will not have an impact on the individual's access to future ECHO programs. Participants will be informed that their participation is voluntary and that responses will remain anonymous and confidential. No names shall be used in any publications or reports of evaluation findings. Project staff will store the signed consent forms in locked cabinets.

### Prerequisites for Implementation and Potential Barriers and Limitations

The following considerations may be a prerequisite to implementing this evaluation: (1) garner political will and engage leadership early to affirm the value of monitoring performance and measuring impact; (2) integrate dedicated funding/resources for evaluation in the workplan and budget of ECHO programming; (3) dedicate personnel (ECHO Champion) who understand the value of evaluation to lead the adoption and implementation of this protocol and monitor performance and measure impact; (4) adapt, contextualize and utilize any or all parts of the comprehensive evaluation framework and tools within a local context; (5) ability to collect, analyze, and manage quantitative and qualitative data (e.g., focus group facilitation, qualitative analysis). Additional specialized skillsets and subject matter expertise may be needed in some settings. Without these prerequisites, we acknowledge that implementing some or all of this protocol may be challenging.

### Anticipated Benefits for Local Partners and ECHO Programs

Information and data gathered from this evaluation will identify essential elements for developing and implementing a high-quality ECHO program. These recommendations will be beneficial for program implementation and evaluation planners irrespective of disease, health condition, or geographic location. Because this evaluation framework is intended to be locally driven, the experience may facilitate the development of local capacity to engage in activities to better monitor, evaluate, and assess program performance beyond ECHO. Moreover, the process monitoring, and evaluation tools may be modified, contextualized, and utilized for other ECHO programs and programmatic activities. Thus, these evaluation activities may serve as a force multiplier (29) to strengthen evaluation capacity, improve public health delivery, and save resources.

## Publications and Dissemination of Results

Findings from this evaluation may be presented at scientific conferences and in peer-reviewed journals. A team consisting of evaluation investigators, including representatives from investigating or collaborating institutions, will be responsible for approving all presentations and publications developed from evaluation data.

## Conclusion

Many countries have been implementing Project ECHO for disseminating best practices to address a variety of disease topics, however evaluation activities have not been consistent or standardized. This protocol offers a comprehensive evaluation framework with a data collection toolkit with the potential to be contextualized and used immediately. This framework offers a unique process to engage a diverse group of stakeholders using an appreciative inquiry process to co-create a comprehensive evaluation framework and a compendium of data collection tools using a mixed methods developmental evaluation approach. Co-creation of data collection tools for an evaluation framework is designed to support action learning from implementation and dissemination, and thus may be worthy of wider use.

## Author Contributions

SG, BBS, EP, and PKM conceived, designed, and drafted the initial manuscript. BMR, IM, EM, EP, and SS provided technical support to develop the toolkit. SG, BMR, IM, EM, JL, EP, SS, BBS, and PKM revised the initial draft and provided critically important intellectual content. SG, BMR, EP, and PKM integrated all feedback and resolved any questions from internal and required clearance at CDC prior to publication. SG and PKM responded to peer-reviewers and revised the manuscript for pre-publication. PKM provided overall leadership and oversight. All authors attest to the accuracy and integrity of final version.

## Funding

This project has been supported by the President's Emergency Plan for AIDS Relief (PEPFAR) through the Centers for Disease Control and Prevention (CDC) under the terms of Cooperative Agreement No: NU2GGH001950-00.

## Author Disclaimer

The findings and conclusions in this publication are those of the authors and do not necessarily represent the official position of the funding agencies, US Centers for Disease Control and Prevention, or the US Government.

## Conflict of Interest

BBS is Associate Director of Project ECHO, of the ECHO Institute, located in Albuquerque, University of New Mexico. The remaining authors declare that the research was conducted in the absence of any commercial or financial relationships that could be construed as a potential conflict of interest.

## Publisher's Note

All claims expressed in this article are solely those of the authors and do not necessarily represent those of their affiliated organizations, or those of the publisher, the editors and the reviewers. Any product that may be evaluated in this article, or claim that may be made by its manufacturer, is not guaranteed or endorsed by the publisher.

## References

[1] The United States President's Emergency Plan for AIDS Relief. Annual Report to Congress. Available online at: https://www.state.gov/wp-content/uploads/2019/09/PEPFAR2019ARC.pdf (accessed October 1, 2019).

[2] UNAIDS AIDSinfo. Available online at: http://aidsinfo.unaids.org/ (accessed September 30, 2019).

[3] Global Health Workforce Alliance. Scaling Up: Saving Lives. World Health Organization, Geneva (2008). Available online at: https://www.who.int/workforcealliance/documents/Global_Health%20FINAL%20REPORT.pdf (accessed May 29, 2020).

[4] World Health Organization. Scaling up Medical and Nursing Education. Report on the WHO/PEPFAR Planning Meeting on Scaling Up Nursing and Medical Education Geneva, 13-14 October. (2009). Available online at: https://www.who.int/hrh/resources/scaling-up_planning_report.pdf (accessed May 29, 2020).

[5] World Health Organization. The World Health Report 2006: Working Together for Health. Geneva: World Health Organization (2006).

[6] Collins FS Glass RI Whitescarver J Wakefield M Goosby EP. Public health. developing health workforce capacity in Africa. Science. (2010) 330:1324–5. 10.1126/science.1199930 21127233 PMC5101931

[7] Narain JP. Public health challenges in India: seizing the opportunities. Indian J Community Med. (2016) 41:85–88. 10.4103/0970-0218.177507 27051080 PMC4799645

[8] World Health Organization. Global Health Observatory. (2016). Available online at: https://www.who.int/data/gho/data/indicators/indicator-details/GHO/medical-doctors-(per-10-000-population) (accessed December 27, 2020).

[9] Struminger B Arora S Zalud-Cerrato S Lowrance D Ellerbrock T. Building virtual communities of practice for health. Lancet. (2017) 390:12–8. 10.1016/S0140-6736(17)31666-5 28816126 PMC6402556

[10] Arora S Kalishman S Thornton K Dion D Murata G Deming P. Expanding access to hepatitis C virus treatment—Extension for Community Healthcare Outcomes (ECHO) project: disruptive innovation in specialty care. Hepatology. (2010) 52:1124–33. 10.1002/hep.23802 20607688 PMC3795614

[11] Meyers DC Katz J Chien V Wandersman A Scaccia JP Wright A. Practical implementation science: developing and piloting the quality implementation tool. Am J Community Psychol. (2012) 50:481–96. 10.1007/s10464-012-9521-y 22618025

[12] Durlak JA. The Importance of Quality Implementation for Research, Practice, and Policy. Washington DC: Office of the Assistant Secretary for Planning and Evaluation, UDDHHS (2013).

[13] Durlak JA DuPre EP. Implementation matters: a review of research on the influence of implementation on program outcomes and the factors affecting implementation. Am J Community Psychol. (2008) 41:327–50. 10.1007/s10464-008-9165-0 18322790

[14] Klein KJ Sorra JS. The challenge of innovation implementation. Acad Manag Rev. (1996) 21:1055–80.

[15] Mbaruku GM Larson E Kimweri A Kruk ME. What elements of the work environment are most responsible for health worker dissatisfaction in rural primary care clinics in Tanzania? Hum Resour Health. (2014) 12:38. 10.1186/1478-4491-12-38 25086596 PMC4131055

[16] Tanzania Ministry of Health. Global AIDS Response Country Progress Report. (2014). (accessed October 1, 2019).

[17] Maxwell J. Qualitative Research Design, an Interactive Approach. 3rd ed. Los Angeles, CA: Sage Publishing (2013).

[18] Stavros JM Hinrichs G. (Eds.). SOARing to high and engaging performance: an appreciative approach to strategy [Special issue]. AI Practitioner Int J Appreciat Inquiry. (2007) 9. Available online at: https://aipractitioner.com/product/ai-practitioner-august-2007/

[19] Gamble JA. A developmental evaluation primer. Montreal, JW: McConnell Foundation (2008).

[20] Patton M. Developmental Evaluation: Applying Complexity Concepts to Enhance Innovation and Use. The Guilford Press l (2011).

[21] Evans R Scourfield J Murphy S. Pragmatic, formative process evaluations of complex interventions and why we need more of them. J Epidemiol Community Health. (2015) 69:925–6. 10.1136/jech-2014-204806 25480407

[22] Ivankova NV Creswell JW Stick S. Using mixed methods sequential explanatory design: From theory to practice. Field Methods. (2006) 18:3–20. 10.1177/1525822X05282260

[23] Ghosh S Singla N Risley K Pinsker E Damle R Struminger B. Co-creating an evaluation framework for tuberculosis extension for community health outcomes (TB-ECHO) model in New Delhi, India. Int J Tuberc Lung Dis. (2018) 22(Suppl. 2):S319.

[24] Paul J. Between-method triangulation in organizational diagnosis. Int J Organiz Analysis. (1996) 4:135–53.

[25] Yin RK. Mixed methods research: parallel or truly integrated? Res Schools. (2006) 13:41–7.

[26] Patton M. Qualitative Research and Evaluation Methods. 3rd ed. Sage Publications (2002).

[27] CDC evaluation framework. Available online at: https://www.cdc.gov/eval/ (accessed October 12, 2018).

[28] Hsieh HF Shannon SE. Three approaches to qualitative content analysis. Qual Health Res. (2005) 15:1277–88. 10.1177/1049732305276687 16204405

[29] Barash D. Project ECHO: Force Multiplier for Community Health Centers. Health Affairs (2015). Available online at: https://www.healthaffairs.org/do/10.1377/hblog20150720.049437/full/ (accessed August 18, 2021).

