# Supplementary material for: A Protocol for a Comprehensive Monitoring and Evaluation Framework With a Compendium of Tools to Assess Quality of Project ECHO (Extension for Community Healthcare Outcomes) Implementation Using Mixed Methods, Developmental Evaluation Design
Source: Front Public Health. 2021 Sep 21;9:714081. doi: 10.3389/fpubh.2021.714081 (PMC8491604; doi:10.3389/fpubh.2021.714081)
Supplement: Supplementary file 1 [file Data_Sheet_1.zip › Appendix 1.docx]

**Appendix 1 : ECHO Participant survey**

***What is this survey? What are the possible risks and benefits to participating in this survey?***

We are interested in your experience with the Tanzania HIV ECHO program. This is an anonymous online survey including 80 questions. This survey is part of a larger effort to pilot test data collection tools for a framework to monitor and evaluate HIV ECHO program implementation, assess program impact through the development of a community of practice, and assess individual satisfaction and learning. You are invited to participate since you have attended at least one HIV ECHO session in 2018 or 2019. Your participation is voluntary. If you choose not to participate, it will not affect your ability to attend current or future ECHO sessions. There are no potential risks to participating in this survey.

The survey will take approximate 30 minutes to complete. You may also save this survey if you are unable to complete it in one sitting and submit after you complete it. You can choose to stop at any time, even if the survey is not complete. We will not collect any personally identifiable information; all information provided will be protected by the survey team. Only members of the independent study team will have access to your responses. We will not share any individual responses with anyone for any reason. Your responses will help inform our recommendations to improve the quality of Tanzania HIV ECHO program and services. An aggregated summary of all participant responses will be available at the conclusion of the analysis. If you have any questions about this survey, or your participation, please contact the study principle investigator:

(Insert PI information)

**Participant Consent:**

**Signature (enter your initials)**

Name of your organization:

By clicking the “Accept” icon below, you are acknowledging you understand the purpose of the survey and accept the potential risks of participating in the survey. Your participation is voluntary. If you choose not to participate, it will not affect your ability to attend current or future ECHO sessions.

**Survey Format and Questions:**

Thank you for agreeing to participate! We deeply appreciate your frank and honest responses to the following questions. Your opinion matters. Your responses will help inform our recommendations to improve the quality of Tanzania HIV ECHO program.

1. **What is your involvement in the HIV ECHO program?**

( ) Participant only

( ) Participant faculty (Personally presented at least one case or didactic material)

( ) ECHO Subject Matter Expert who has facilitated at least one case presentation or presented a didactic presentation

1. **Where do you typically attend ECHO sessions?**

( ) In an enclosed space (e.g., walled office, meeting room, conference hall)

( ) In an open space (e.g. cubicle, other structure without a door)

1. **When you attend ECHO sessions at your facility, how many persons accompany you in the same room?**

( ) I attend alone

( ) 1 – 5 people

( ) 6 – 10 people

( ) 11 – 15 people

( ) more than 15 people

1. **What equipment do you typically use when participating in ECHO sessions?**

( ) Professionally-installed video conferencing equipment (e.g. high-quality web-camera, high-definition screen)

( ) Video-enabled computer with external projection screen

( ) Video-enabled computer individual screen

( ) Computer without video capability (i.e., I can see and hear session material, but others cannot see me)

( ) Dial in using a smart phone (i.e., I can see and hear session material, and others cannot see me)

( ) Dial in using a phone (i.e., listening only; I cannot see material; others cannot see me)

1. **Approximately how many ECHO sessions have you attended?**

( ) 1

5b.) Please share why you were not able to join more than 1 session:

( ) My facility just joined ECHO

( ) Not interested

( ) Not applicable to my work

( ) No time to participate

( ) No equipment to connect

( ) Not supported by my boss

( ) Other: ______________________________

( ) 2-5

( ) 6 – 10

( ) 11 – 20

( ) 21 – 50

( ) >50

( ) I never missed a single session! : )

1. **Please identify the challenges that keep you from attending the HIV ECHO sessions. (Check all that apply)**

( ) Busy patient schedule

( ) Other professional activities such as conferences, lectures

( ) Family commitments

( ) Traffic delays during the commute to work

( ) I forget/I do not receive reminders

( ) Other __________________

Please describe and comment on ways to address the barriers, if any, that you encounter:

___________________________________________________________________________

1. **What best describes your current position? (Choose only one)**
2. ( ) Physician
3. ( ) Medical officer
4. ( ) Assistant medical officer
5. ( ) Clinical officer
6. ( ) Assistant clinical officer
7. ( ) Medical attendant
8. ( ) Registered nurse
9. ( ) Enrolled nurse
10. ( ) Assistant nursing officer
11. ( ) Pharmacist
12. ( ) Pharmacy technician
13. ( ) Laboratory assistant
14. ( ) Laboratory technician
15. ( ) Assistant laboratory technician
16. ( ) Physiotherapist
17. ( ) Peer educator
18. ( ) Data officer
19. ( ) Community health worker
20. ( ) Information technology
21. ( ) Student
22. **For how long have you been working in this position?**

( ) Less than one year

( ) 1 – 5 years

( ) 6 – 10 years

( ) More than 10 years

1. **For how long have you been working in the field of HIV care and treatment?**

( ) Less than one year

( ) 1 – 5 years

( ) 6 – 10 years

( ) More than 10 years

1. **How did you obtain your HIV education and training? (Check all that apply)**

( ) Medical or Nursing College

( ) On-the-job training

( ) Instructor-led seminars and workshops

( ) Self-study modules

( ) Online coursework

( ) Clinical mentor

( ) Professional conferences and meetings

( ) Other; please describe: ________________________________

**11. Have you obtained specialized training for HIV treatment, prevention, and care in the last 2 years, aside from participation in the HIV ECHO program?**

( ) Yes

( ) No

11 b.) If yes, please provide information about the name/type and length of the training, and the name of the institution that provided the training. _______________________________________________________________________

**12. What best describes your level of supervision?**

( ) I have no supervisory responsibilities

( ) I supervise others

**13. What best describes your professional practice?**

( ) Public practice only

( ) Private practice only

( ) Both public and private practice

**14. On an average day, how many HIV patients do you provide service for?**

( ) None

( ) 1 – 5

( ) 6 – 10

( ) 11 – 20

( ) 21 - 50

( ) 50 – 100

( ) 100+

**15. Do you have access to a video-enabled computer or laptop that you can use regularly?**

( ) Yes, computer/laptop

( ) No

**16. Do you own or have access to a smart phone or tablet that you can use regularly?**

( ) Yes

( ) No

**17. At your institution, do you have access to the following facilities (Check all that apply):**

( ) Professionally-installed video conferencing equipment (e.g. high-quality web-camera, high-definition screen)

( ) Video-enabled computer with external projection screen

( ) Video-enabled computer individual screen

( ) Computer without video capability (i.e., I can see and hear session material, but others cannot see me)

( ) I don’t have access to technology at my institution, I travel to other places to participate in ECHO

**18. Check the option that best applies to your interaction with computers**

( ) I have very little or no experience with computers (no experience)

( ) I can operate computers with assistance from others (novice user)

( ) I can operate without assistance from others (average user)

( ) I am the one usually assisting others who have trouble with computers (expert user)

**19. How often do you check email?**

( ) I do not use/check email

( ) At least monthly

( ) Once or twice week

( ) At least once a day

( ) At least once an hour

( ) As soon as I receive a message

**20. Have you ever participated in an on-line or distance learning course other than HIV ECHO?**

( ) Yes

( ) No

( ) Not sure/Don’t know

**21. In the past 12 months, how did you access professional development activities? (Check all that apply)**

( ) Attending in-person training within my facility

( ) Attending virtual trainings such as ECHO within my facility

( ) Attending virtual trainings such as ECHO outside my facility but within my region, so I have to travel off-site

( ) Attending in-person courses outside my facility but within my region, so I have to travel off-site

( ) Traveling outside my region to attend in-person trainings/courses/lectures

( ) Comment on other trainings attended ________________________________________________

**Please reflect on professional development, learning in the context of the HIV ECHO sessions that you attended. Choose the option that best reflects your opinion.**

**22. I attend ECHO sessions to learn new information**

( ) Strongly Agree ( ) Agree ( ) Not sure ( ) Disagree ( ) Strongly Disagree

**23. I attend ECHO sessions to interact with my colleagues**

( ) Strongly Agree ( ) Agree ( ) Not sure ( ) Disagree ( ) Strongly Disagree

**24. I attend ECHO sessions because I am required to participate**

( ) Strongly Agree ( ) Agree ( ) Not sure ( ) Disagree ( ) Strongly Disagree

**25. I attend ECHO sessions because my institution/supervisor asked me to represent them**

( ) Strongly Agree ( ) Agree ( ) Not sure ( ) Disagree ( ) Strongly Disagree

**26. I attend ECHO sessions for my personal growth**

( ) Strongly Agree ( ) Agree ( ) Not sure ( ) Disagree ( ) Strongly Disagree

1. **I attend ECHO sessions because my colleagues attend it**

( ) Strongly Agree ( ) Agree ( ) Not sure ( ) Disagree ( ) Srongly Disagree

1. **I usually learn something new during an ECHO session**

( ) Strongly Agree ( ) Agree ( ) Not sure ( ) Disagree ( ) Strongly Disagree

1. **ECHO sessions are repetitive**

( ) Strongly Agree ( ) Agree ( ) Not sure ( ) Disagree ( ) Strongly Disagree

1. **During ECHO sessions, Subject Matter Experts (SMEs) and participants are equals**

( ) Strongly Agree ( ) Agree ( ) Not sure ( ) Disagree ( ) Strongly Disagree

1. **ECHO faculty are well-prepared for each session**

( ) Strongly Agree ( ) Agree ( ) Not sure ( ) Disagree ( ) Strongly Disagree

1. **I feel comfortable asking questions during ECHO sessions**

( ) Strongly Agree ( ) Agree ( ) Not sure ( ) Disagree ( ) Strongly Disagree

1. **ECHO faculty are engaging and encourage interactions between participants during each session**

( ) Strongly Agree ( ) Agree ( ) Not sure ( ) Disagree ( ) Strongly Disagree

1. **I feel I can contact ECHO SMEs outside of ECHO sessions for clinical support or assistance**

( ) Strongly Agree ( ) Agree ( ) Not sure ( ) Disagree ( ) Strongly Disagree

1. **ECHO sessions take away time from other important activities**

( ) Strongly Agree ( ) Agree ( ) Not sure ( ) Disagree ( ) Strongly Disagree

1. **Since participating in ECHO, I am more likely to discuss my HIV clinical experience with my colleagues on a regular basis**

( ) Strongly Agree ( ) Agree ( ) Not sure ( ) Disagree ( ) Strongly Disagree

1. **I enjoy learning from the experiences of my peers**

( ) Strongly Agree ( ) Agree ( ) Not sure ( ) Disagree ( ) Strongly Disagree

1. **HIV ECHO has improved my confidence in treating HIV patients**

( ) Strongly Agree ( ) Agree ( ) Not sure ( ) Disagree ( ) Strongly Disagree

1. **The short presentations (termed as didactic portions) during the ECHO sessions are an effective way for me to learn about best practices in patient care**

( ) Strongly Agree ( ) Agree ( ) Not sure ( ) Disagree ( ) Strongly Disagree

1. **The didactic presentations during the HIV ECHO sessions provide me with useful up-to-date knowledge**

( ) Strongly Agree ( ) Agree ( ) Not sure ( ) Disagree ( ) Strongly Disagree

1. **The case-based discussions during the HIV ECHO sessions are relevant to my clinical practice**

( ) Strongly Agree ( ) Agree ( ) Not sure ( ) Disagree ( ) Strongly Disagree

1. **I have changed my treatment plan for at least one patient based on the information learned during a HIV ECHO session**

( ) Strongly Agree ( ) Agree ( ) Not sure ( ) Disagree ( ) Strongly Disagree

1. **I use the knowledge gained during HIV ECHO sessions to improve the quality of care of my patients**

( ) Strongly Agree ( ) Agree ( ) Not sure ( ) Disagree ( ) Strongly Disagree

1. **I think my patients are benefiting from what I learn during ECHO sessions**

( ) Strongly Agree ( ) Agree ( ) Not sure ( ) Disagree ( ) Strongly Disagree

1. **Please describe how your patients benefit from what you learn during ECHO sessions:**
2. **I am interested in joining other Project ECHO sessions outside of HIV**

( ) Strongly Agree ( ) Agree ( ) Not sure ( ) Disagree ( ) Strongly Disagree

**Please reflect on logistics of ECHO sessions that you have attended. Choose the option that best fits your opinion.**

1. **Which segment of the HIV ECHO sessions do you like the most? (Select best option)**

( ) Didactic lecture presentation

( ) Case presentation

( ) Discussion

( ) No preference

1. **The HIV ECHO sessions usually last about an hour. What do you think about the length of each session?**

( ) Too long ( ) Just right ( ) Too short

1. **Please circle two best days/times (AM/PM) you would like to attend the ECHO sessions.**

( ) Monday AM ( ) Monday PM ( ) Tuesday AM ( ) Tuesday PM

( ) Wednesday AM ( ) Wednesday PM ( ) Thursday AM ( ) Thursday PM

( ) Friday AM ( ) Friday PM

1. **Please suggest session topics that would be helpful for your clinical practice.**

TOPICS:

1. __________________ 2. _________________ 3. __________________ 4. ___________________

**51. How frequently do you share the information from HIV ECHO sessions with colleagues who are unable to attend ECHO sessions? (Select best option)**

( ) Always ( ) Frequently ( ) Sometimes ( ) Seldom ( ) Never

**52. How frequently would you like the HIV ECHO sessions to take place?**

( ) Once a week

( ) Once a month

( ) Twice a month

( ) Quarterly

( ) Other____________

**53. Do you think HIV ECHO sessions should continue?**

( ) Yes ( ) No ( ) Not sure

53 b.) Please explain why not/not sure: _____________________________________________

**When you think of high-quality ECHO implementation, choose the option that best reflects your opinion.**

**54. My institute has ECHO champions who support my participation**

( ) Strongly agree ( ) Agree ( ) Not sure ( ) Disagree ( ) Not Applicable/Don’t know

**55. MOH has leadership support to improve HIV ECHO implementation**

( ) Strongly agree ( ) Agree ( ) Not sure ( ) Disagree ( ) Not Applicable/Don’t know

**56. UMB has leadership support to improve HIV ECHO implementation**

( ) Strongly agree ( ) Agree ( ) Not sure ( ) Disagree ( ) Not Applicable/Don’t know

**57. NACP has leadership support to improve HIV ECHO implementation**

( ) Strongly agree ( ) Agree ( ) Not sure ( ) Disagree ( ) Not Applicable/Don’t know

**58. UMB requires external support to implement the HIV ECHO programme**

( ) Strongly agree ( ) Agree ( ) Not sure ( ) Disagree ( ) Not Applicable/Don’t know

**59. MOH has stable and sustainable funding support to implement HIV ECHO**

( ) Strongly agree ( ) Agree ( ) Not sure ( ) Disagree ( ) Not Applicable/Don’t know

**60. UMB has stable and sustainable funding support to implement HIV ECHO**

( ) Strongly agree ( ) Agree ( ) Not sure ( ) Disagree ( ) Not Applicable/Don’t know

**61. MOH provides technical assistance to help me manage my complicated HIV cases**

( ) Strongly agree ( ) Agree ( ) Not sure ( ) Disagree ( ) Not Applicable/Don’t know

**62. UMB provides technical assistance to help me manage my complicated HIV cases**

( ) Strongly agree ( ) Agree ( ) Not sure ( ) Disagree ( ) Not Applicable/Don’t know

**63. MOH encourages HIV ECHO participation from a variety of stakeholders, such as private colleges, private hospitals or other partners outside the public sector**

( ) Strongly agree ( ) Agree ( ) Not sure ( ) Disagree ( ) Not Applicable/Don’t know

**64. UMB encourages HIV ECHO participation from a variety of stakeholders, such as private colleges, private hospitals or other partners outside the public sector**

( ) Strongly agree ( ) Agree ( ) Not sure ( ) Disagree ( ) Not Applicable/Don’t know

**65. HIV ECHO sessions demonstrate commitment towards HIV epidemic control, through improved treatment outcomes of complicated HIV or HIV-TB co-infected cases**

( ) Strongly agree ( ) Agree ( ) Not sure ( ) Disagree ( ) Not Applicable/Don’t know

**66. MOH has appropriate logistics and infrastructure to coordinate and expand ECHO sessions to all districts in Tanzania**

( ) Strongly agree ( ) Agree ( ) Not sure ( ) Disagree ( ) Not Applicable/Don’t know

**67. UMB has appropriate logistics and infrastructure to coordinate and expand ECHO sessions to all facilities in Tanzania**

( ) Strongly agree ( ) Agree ( ) Not sure ( ) Disagree ( ) Not Applicable/Don’t know

**68. UMB documents and follows up with recommendations that were shared during HIV ECHO sessions**

( ) Strongly agree ( ) Agree ( ) Not sure ( ) Disagree ( ) Not Applicable/Don’t know

**69. Results from quarterly surveys at ECHO sessions inform ECHO implementation**

( ) Strongly agree ( ) Agree ( ) Not sure ( ) Disagree ( ) Not Applicable/Don’t know

**70. UMB has effective communication strategies in place to maintain support of partners**

( ) Strongly agree ( ) Agree ( ) Not sure ( ) Disagree ( ) Not Applicable/Don’t know

**71. MOH has effective communication strategies in place to maintain support of partners**

( ) Strongly agree ( ) Agree ( ) Not sure ( ) Disagree ( ) Not Applicable/Don’t know

**72. UMB’s goals and objective for ECHO are clear to all stakeholders**

( ) Strongly agree ( ) Agree ( ) Not sure ( ) Disagree ( ) Not Applicable/Don’t know

**73. MOH’s goals and objective for ECHO are clear to all stakeholders**

( ) Strongly agree ( ) Agree ( ) Not sure ( ) Disagree ( ) Not Applicable/Don’t know

**74. UMB has long-term vision and plans for ECHO that are shared with all stakeholders**

( ) Strongly agree ( ) Agree ( ) Not sure ( ) Disagree ( ) Not Applicable/Don’t know

**75. MOH has long-term vision and plans for ECHO that are shared with all stakeholders**

( ) Strongly agree ( ) Agree ( ) Not sure ( ) Disagree ( ) Not Applicable/Don’t know

**76. In your opinion, are there other considerations that make ECHO implementation high quality? Please list 3 important ones that come to mind:**

1.)

2.)

3.)

**77. Please answer the following questions about yourself:**

**Gender:** ( ) Male ( ) Female ( ) Rather not share

**78. Date survey completed: dd/mm/yy**

**79. State where you join HIV ECHO sessions:**

**80. Anything else you would like to share with us that you think will help improve the Tanzania HIV ECHO program?**

ASANTE FOR PARTICIPATING IN THIS SURVEY!
