# Supplementary material for: A Protocol for a Comprehensive Monitoring and Evaluation Framework With a Compendium of Tools to Assess Quality of Project ECHO (Extension for Community Healthcare Outcomes) Implementation Using Mixed Methods, Developmental Evaluation Design
Source: Front Public Health. 2021 Sep 21;9:714081. doi: 10.3389/fpubh.2021.714081 (PMC8491604; doi:10.3389/fpubh.2021.714081)
Supplement: Supplementary file 1 [file Data_Sheet_1.zip › Appendix 2A-C.docx]

**Appendix 2A: Focus Group Discussion Guide for Facility HIV ECHO Health Care Providers (HCPs)**

| **Date** | **\|___\|\|___\| / \|___\|\|___\| / \|___\|\|___\|*(mm/dd/yy)*** |
| --- | --- |
| **Facilitator initials** |  |
| **Co-facilitator initials** |  |
| **Start Time** | **\|___\|\|___\| : \|___\|\|___\|*(hour/min)*** |
| **End Time** | **\|___\|\|___\| : \|___\|\|___\|*(hour/min)*** |
| **Number of participants** | **\|___\|\|___\|** |

Good morning/afternoon/evening. My name is _______________. I am from _______________ and am here to learn from you about the HIV ECHO program. My co-facilitator ________________ is also with us today and will be assisting with our discussion and taking notes. We will also be audio recording the sessions. I would like to assure you that all responses and results will remain anonymous when we report the findings, so feel free to share openly and honestly. We ask all participants to respect the views and perspectives of their colleagues. There are no “right” or “wrong” answers, and everyone’s opinions are equally valuable to our study. Please refrain from interrupting others while they are speaking. If you want to add to a particular point, please raise your hand, I will do my best to call on you at an appropriate time. We ask that all present not share items discussed in the focus group outside the group.

**There have been over 50 HIV ECHO sessions to date, please share:**

1. **Approximately how many ECHO sessions have you attended?**
2. **Why do you participate in the HIV ECHO sessions? [7–10 mins]**
3. **Please think about the last ECHO session you attended [20–30 mins]:**

a) How well did the brief lecture address your needs?

b) In what ways do you see yourself using the knowledge you learned in the lectures?

c) What do you think about the content, relevance, usefulness, and clarity of the presentation?

d) How do you think what you learned may be applicable in patient care and management?

e) Can you think of your favorite part of the lecture? What did you like most about it?

f) Can you give an example of one of the least favorite parts of the didactic presentation? What did you not like about it?

**4. Please think about the last case study or didactic presentation you gave to share your expertise:**

**a) What did you present? Case study or didactic presentation?**

**b) How long before your presentation were you contacted by UMB staff? Was this sufficient time to prepare?**

**c) How much time and effort did it take for you to prepare for the presentation?**

**d) Were you given any guidance or pointers on how to prepare your presentations? If so, please explain.**

**e) Were you able to include learning objectives for your didactic presentations?**

**f) For didactic presentations, what did you do to ensure high-quality course content?**

**g) How did you interact or engage with your audience during the ECHO session?**

**4. Were you asked any questions DURING the session?**

1. **Did anyone reach out to you with questions or clarifications AFTER your presentation session?**

**6. Did you have a favorite part of the presentation? If yes, what? If no, why not?**

**7. Did you have a least favorite part of the didactic presentation? What did you not like about it?**

**8. How do you suggest recommendations that either you or others provide at these ECHO sessions be documented? How were your recommendations documented and shared?**

**9. In your opinion, where do you think these recommendations could be documented? Stored? How should they be shared and with whom?**

**10. Should it be anyone’s responsibility to document or follow-up with the recommendations that are provided at these ECHO sessions? If yes, who?**

**11. How often should there be follow-up in your opinion?**

**12.What words of advice do you have for a colleague who is planning to present a didactic presentation at an upcoming ECHO session?**

**13. What words of advice do you have for your UMB colleagues who are coordinating these ECHO sessions to implement high-quality HIV ECHO sessions?**

**14. As you know, there are plans to expand HIV ECHO to cover a larger number of facilities in Tanzania and/or becoming a super hub with zonal or regional hubs. In your opinion from your current experience, what are the 3 most important things that should be prioritized as this program is scaled up?**

**1.**

**2.**

**3.**

**15. Do you feel HIV ECHO may play a role in Tanzania’s plan towards HIV-TB epidemic control? If yes, how? If not, why not?**

**16. Do you have additional comments or suggestions that you would like to share about HIV ECHO?**

**Appendix 2B: Focus Group Guide for HIV ECHO session participants**

| **Date** | **\|___\|\|___\| / \|___\|\|___\| / \|___\|\|___\|***(mm/dd/yy)* |
| --- | --- |
| **Facilitator initials** |  |
| **Co-facilitator initials** |  |
| **Start Time** | **\|___\|\|___\| : \|___\|\|___\|***(hour/min)* |
| **End Time** | **\|___\|\|___\| : \|___\|\|___\|***(hour/min)* |
| **Number of participants** | **\|___\|\|___\|** |

Good morning/afternoon/evening. My name is _______________. I am from ____________and am here to learn from you about the ECHO program. My co-facilitator ________________ is also with us today and will be assisting with our discussion and taking notes. We will also be audio recording the sessions. I would like to assure you that all responses and results will remain anonymous when we report the findings, so feel free to share openly and honestly. We ask all participants to respect the views and perspectives of their colleagues. There are no “right” or “wrong” answers, and everyone’s opinions are equally valuable to our study. Please refrain from interrupting others while they are speaking. If you want to add to a particular point, please raise your hand, I will do my best to call on you at an appropriate time. We ask that all present not share items discussed in the focus group outside the group.

[Ice breaking exercise]: Before we start, let us go around the session and introduce ourselves and tell us 1 favorite place you have visited recently and why? [5 –10 mins]

**There have been over 50 ECHO sessions to date, please share:**

**1. Why do you participate in the HIV ECHO sessions? [7–10 mins]**

**2. Please think about the last ECHO session you attended [20–30 mins]:**

a) How well did the brief lecture address your needs?

b) In what ways do you see yourself using the knowledge you learned in the lectures?

c) What do you think about the content, relevance, usefulness, and clarity of the presentation?

d) How do you think what you learned may be applicable?

e) Can you think of your favorite part of the lecture? Why?

f) Can you give an example of one of the least favorite parts of the didactic presentation? What did you not like about it?

**3. Please think about a recent patient case presentation where your colleagues presented [20–30 mins].**

a) Are the case presentations applicable to your practice? Do you encounter similar cases?

b) How well does the case presentation and recommendation format address your needs to learning about HIV patient treatment, care and management?

c) Have you used what you have learned from the ECHO session? In what ways did you use what you learn from the case-scenarios?

c) What do you think about the content, relevance, usefulness, and clarity of the case-scenario presentations?

d) Can you think of your favorite case scenario presentation? What did you like most about it?

e) Can you give an example of one of your least favorite case presentations? What did you not like as much?

f) In your opinion, what could be improved in the case presentations and discussions?

**4. Do you feel comfortable asking questions during the ECHO clinic? If no, why not? How do you think this could be improved? [7–10 mins]**

**5. How have you been able to apply concepts or knowledge you learned during the HIV ECHO sessions to patients with similar problems in your practice? [7–10 mins]**

**6. How do you feel participating in the UMB HIV ECHO program has changed the way you work? Manage patients? If so, how? If not, why not?**

**7. How do you share “lessons learned” from HIV ECHO sessions with others colleagues? [15–20 mins]**

a) Please describe what facilitates or inhibits sharing information and practices that you learn at HIV ECHO with your colleagues

Probe: What forum do you use to share information with your team or other clinical staff in your health care facility? What are the obstacles in sharing information?

b) Do you think other providers in your clinic would benefit from participation in the HIV ECHO?

**8. What do you see are the advantages and disadvantages of the current ECHO sessions? [7–10 mins]**

**9. How do you document the recommendations? [7–10 mins]**

**10. In your opinion, where do you think these recommendations should be documented? And followed-up on? How often should there be follow-up? Who should be responsible for follow up in your opinion? [10–12 mins]**

**11. As you know, there are plans to expand HIV ECHO to states all over India. In your opinion from your current experience, what are the 3 most important things that should be considered or prioritized as this program is expanded? [15 – 20 mins]**

**1.**

**2.**

**3.**

**12. If you could redesign HIV ECHO? What aspects would you change? What aspects would you improve? What aspects would you remove? What aspects would you keep? [7–10 mins]**

**13. Do you feel HIV ECHO may play a role in HIV epidemic control in Tanzania? If yes, how? If no, why not? [7–10 mins]**

**14. Do you have additional comments or suggestions that you would like to share? [5–7 mins]**

**Appendix 2C: Focus Group Discussion Guide for TB ECHO Didactic/Case presenters/Subject Matter Experts**

| **Date** | **\|___\|\|___\| / \|___\|\|___\| / \|___\|\|___\|*(mm/dd/yy)*** |
| --- | --- |
| **Facilitator initials** |  |
| **Co-facilitator initials** |  |
| **Start Time** | **\|___\|\|___\| : \|___\|\|___\|*(hour/min)*** |
| **End Time** | **\|___\|\|___\| : \|___\|\|___\|*(hour/min)*** |
| **Number of participants** | **\|___\|\|___\|** |

Good morning/afternoon/evening. My name is _______________. I am from ___________ and am here to learn from you about the ECHO program. My co-facilitator ________________ is also with us today and will be assisting with our discussion and taking notes. We will also be audio recording the sessions. I would like to assure you that all responses and results will remain anonymous when we report the findings, so feel free to share openly and honestly. We ask all participants to respect the views and perspectives of their colleagues. There are no “right” or “wrong” answers, and everyone’s opinions are equally valuable to our study. Please refrain from interrupting others while they are speaking. If you want to add to particular point, please raise your hand, I will do my best to call on you at an appropriate time. We ask that all present not share items discussed in the focus group outside the group.

**There have been over 50 ECHO sessions to date, please share:**

1. **Approximately how many ECHO sessions have you attended?**
2. **Approximately how many patient case/didactic presentations have you prepared and presented?**

**3. Please think about the last patient case presentation or didactic presentation you gave to share your expertise:**

**a) What did you present? Patient case or didactic presentation?**

**b) How long before your presentation were you contacted by UMB staff? Was this sufficient time to prepare?**

**c) How much time and effort did it take for you to prepare for the presentation?**

**d) Were you given any guidance or pointers on how to prepare your presentations?**

**e) Were you able to include learning objectives for your didactic presentations?**

**f) For didactic presentations, what did you do to ensure high-quality course content?**

**g) How did you interact or engage with your audience during the ECHO session?**

**4. Were you asked any questions DURING the session?**

1. **Did anyone reach out to you with questions or clarifications AFTER your presentation session?**

**6. Can you think of your favorite part of the presentation? Why? What did you like and dislike about this learning model? Thoughts about connecting via videoconference, thoughts about the case-study approach, thoughts about the brief didactic approach, thoughts about the community of practice interactive learning approach?**

**7. Can you give an example of one of the least favorite part of the didactic presentation? What did you not like about it?**

**8. How do you suggest recommendations that either you or others provide at these ECHO sessions be documented?**

**9. In your opinion, where do you think these recommendations should be documented? Stored? Shared with whom?**

**10. Should it be someone’s responsibility to document or follow-up with the recommendations that are provided at these ECHO sessions? If so, who?**

**11. How often should there be follow-up in your opinion?**

**12.What words of advice do you have for your colleague who is planning to present the didactic presentation at the next ECHO session?**

**13. What words of advice do you have for your UMB partners who are coordinating these ECHO sessions to implement high-quality ECHO sessions?**

**14. As you know, there are plans to expand HIV ECHO to larger number of spokes, Tanzania ECHO becoming a super hub, scaling up to zonal/regional hubs. In your opinion from your current experience, what are the 3 most important things that should be prioritized as this program is expanded?**

**1.**

**2.**

**3.**

**15. Do you feel HIV ECHO may play a role in HIV epidemic control in Tanzania? If yes, why? If no, why not?**

**16. Do you have additional comments or suggestions that you would like to share?**
