# Supplementary material for: A Protocol for a Comprehensive Monitoring and Evaluation Framework With a Compendium of Tools to Assess Quality of Project ECHO (Extension for Community Healthcare Outcomes) Implementation Using Mixed Methods, Developmental Evaluation Design
Source: Front Public Health. 2021 Sep 21;9:714081. doi: 10.3389/fpubh.2021.714081 (PMC8491604; doi:10.3389/fpubh.2021.714081)
Supplement: Supplementary file 1 [file Data_Sheet_1.zip › Appendix 3.docx]

**Appendix 3: HIV ECHO Focus Group Consent Form**

**Introduction**

The HIV ECHO offers an opportunity to build bridges between MOH, UMB, CDC Tanzania, and local community health partners in an ‘all teach, all learn’ interactive format. To understand the impact of HIV ECHO, the Centers for Disease Control and Prevention (CDC) are developing an evaluation framework and pilot testing data collection tools. We invite you to participate in this focus group because you previously attended the HIV ECHO sessions. We are interested in learning about your experiences and opinions about HIV ECHO sessions you attended.

**What will happen if I decide to participate?**

If you agree to participate, you will attend a focus group discussion conducted by a focus group facilitator. There will be 6-8 participants in each focus group, some of whom you may or may not know. Other evaluation staff will also be attend to help facilitate and record information during the focus group. Focus groups may last from 60-90 minutes.

**What are the risks or side effects of being in this focus group?**

There are minimal risks of discomfort when sharing your opinions and experiences. There is a possible loss of privacy and confidentiality associated with participating in a focus group. There is no way to protect privacy from other participants in the focus group, but everyone participating shall be asked to maintain confidentiality. Discussions about sensitive personal information shall be discouraged.

**What are the benefits to being in this focus group?**

By participating in a focus group, you will be helping us better understand the elements needed for high-quality HIV ECHO implementation. The outcome of our work will produce a systematic and comprehensive process for monitoring and evaluating ECHO programs.

**How will my information be kept confidential?**

There is no way to protect privacy from other participants in the focus group, but everyone participating will be asked to maintain confidentiality. Focus group conversations will be digitally recorded and transcribed with names removed from transcriptions. These recordings that will remain in a locked, secure location within CDC office until they are destroyed at study completion. Your name will not be used in any published reports about this evaluation.

**Can I stop being in the focus group once I begin?**

Your participation is completely voluntary. You have the right to choose not to participate or to withdraw your participation at any point during this interview. Non-participation or withdrawal will NOT affect your employment or participation in future HIV ECHO activities.

**Whom can I call with questions or complaints about this focus group?**

If you have any questions, concerns or complaints at any time, please contact the study Principle Investigator:

(PI Name)

**CONSENT**

I agree to participate in this focus group discussion. I acknowledge the potential risk and benefits of participating, and understand that I may withdraw at any time.

_____________________________________ ___________________

Participant, Name [Print], Signature Date

_____________________________________________________________

Investigator, Name [Print], Signature Date

_____________________________________________________________

Witness, Name [Print], Signature Date
