# Supplementary material for: A Protocol for a Comprehensive Monitoring and Evaluation Framework With a Compendium of Tools to Assess Quality of Project ECHO (Extension for Community Healthcare Outcomes) Implementation Using Mixed Methods, Developmental Evaluation Design
Source: Front Public Health. 2021 Sep 21;9:714081. doi: 10.3389/fpubh.2021.714081 (PMC8491604; doi:10.3389/fpubh.2021.714081)
Supplement: Supplementary file 1 [file Data_Sheet_1.zip › Appendix 4A-C.docx]

**Appendix 3A: ECHO Session Facilitation Scorecard to completed by Objective Reviewer after viewing each of the 3 session recordings**

**Date: _________________________ Observer/Scorer: ____________________________________**

**ECHO Session Date:____________________ Facilitator Name: _____________________________________**

| \| Please rate the following statements based on the observed session:  1 = Never  2 = Rarely  3 = Sometimes  4 = Often  5 = Always  9 = Not Applicable \| \| --- \| | **Never** | **Rarely** | **Sometime** | **Often** | **Always** | **Not Applicable** |
| --- | --- | --- | --- | --- | --- | --- | --- |
| 1.Hub facilitator identifies himself/herself |  |  |  |  |  |  |
| 2.Starts clinic on time (5 minutes flexibility) |  |  |  |  |  |  |
| 3.Hub facilitator ensures that all faculty: didactic and/or case presenter introduce themselves |  |  |  |  |  |  |
| 4. Identifies participants as they sign in |  |  |  |  |  |  |
| 5. Hub facilitator reminds participants to maintain confidentiality (HIPAA) – uses ECHO ID for case presentations |  |  |  |  |  |  |
| 6. Hub facilitator briefly reviews agenda |  |  |  |  |  |  |
| 7. Learning objectives for the session were clearly stated. |  |  |  |  |  |  |
| 8.Eliminates environmental distractions (avoids side conversations, rustling of papers, whispering, unnecessary gestures) |  |  |  |  |  |  |
| 9.The facilitator engages all group members |  |  |  |  |  |  |
| 10.Invites others to share experiences |  |  |  |  |  |  |
| 11.Summarizes patient case presentation in 5 to 6 sentences |  |  |  |  |  |  |
| 12.Provides evidenced-based peer-reviewed publications or national/international guidelines as needed to support recommendations given or to support discussion |  |  |  |  |  |  |
| 13. Shares his/her own experience in relation to the topic or case presented |  |  |  |  |  |  |
| 14.Ensures some HIV related concepts or topics are imparted when providing recommendations |  |  |  |  |  |  |
| 15.Requests feedback from participants who are attending clinic via telephone and video |  |  |  |  |  |  |
| 16.Is encouraging and never makes negative, offensive, or disrespectful comments |  |  |  |  |  |  |
| 17. Encourages participants to introduce themselves prior to speaking |  |  |  |  |  |  |
| 18.Encourages participation by asking open-ended questions |  |  |  |  |  |  |
| 19.The facilitator keeps the clinic on track by managing time, providing coaching or guidance as needed |  |  |  |  |  |  |
| 20.Facilitator gently re-directs when someone is critical or confrontational to a colleague |  |  |  |  |  |  |
| 21.Hub facilitator is supportive, engaging, and listens to peers |  |  |  |  |  |  |
| 22.Creates a supportive learning environment by allowing participants the opportunity to answer questions and contribute to the discussion |  |  |  |  |  |  |
| 23. When addressing the participants, he/she faces the camera and makes eye contact. |  |  |  |  |  |  |
| 24. He/she uses clear and simple language |  |  |  |  |  |  |
| 25.Uses inappropriate responses as teachable moments |  |  |  |  |  |  |
| 26. Facilitator repeats questions/comments asked by participants when needed |  |  |  |  |  |  |

Additional comments from observations:

**Appendix 3B – Didactic Content Review by Objective Reviewers**

**Please complete after viewing each of the 3 HIV ECHO sessions**

**Name of Reviewer: ____________________________ Date Review Completed: ____________________**

**Presenter’s Name: _____________________________ ECHO Session Name: _________________________**

**Date of ECHO Session:______________Start of review time: __________End of review time: _______________**

| \| Please rate the following statements based on the observed session:  1 = Strongly Disagree  2 = Disagree  3 = Not sure  4 = Agree  5 = Strongly Agree \| \| --- \| | | | | **Strongly Disagree** | **Disagree** | **Not sure** | | **Agree** | **Strongly Agree** | **Not Applicable** |
| --- | --- | --- | --- | --- | --- | --- | --- | --- | --- | --- | --- |
| **Applicability and Clarity** | | | |  |  |  | |  |  |  |
| 1. The presentation learning objectives were clearly stated | | | |  |  |  | |  |  |  |
| 1. The presentation was delivered simply and clearly | | | |  |  |  | |  |  |  |
| 1. The slide content was free from errors | | | |  |  |  | |  |  |  |
| 1. The presenter’s narration was easy to follow | | | |  |  |  | |  |  |  |
| 1. The presenter’s narration matched the slide content | | | |  |  |  | |  |  |  |
|  | | | |  |  |  | |  |  |  |
| **Content validity** | | | |  |  |  | |  |  |  |
| 1. Didactic material was consistent with national/international standards of care | | | |  |  |  | |  |  |  |
| 1. Didactic material was timely to current national/international standards of care | | | |  |  |  | |  |  |  |
| 1. References and systematic evidence were cited as evidence for the Didactic material. | | | |  |  |  | |  |  |  |
| **Stakeholder Engagement** | | | |  |  |  | |  |  |  |
| 1. Responses to comments and questions from the audience were accurate and appropriate | | | |  |  |  | |  |  |  |
| 10.) The presenter was effective at engaging the audience | | | |  |  |  | |  |  |  |
| 11.) The presenter appeared well-prepared | | | |  |  |  | |  |  |  |
| 12.) The learning objectives were achieved | | | |  |  |  | |  |  |  |
|  | **Very poor** | **Poor** | **Fair** | **Good** | | | **Excellent** | | |  |
| **13.) Rate the overall quality of this didactic session** |  |  |  |  | | |  | | |  |

**Additional comments from observations:**

**Appendix 3C – Recommendation Review by Objective Reviewers**

**Please complete after viewing each of the 3 HIV ECHO sessions**

**Name of Reviewer: ____________________________ Date Review Completed: _______________________**

**Presenter’s Name: _____________________________ ECHO Session Name: _________________________**

**Date of ECHO session:_____________Start of review time: __________ End of review time: _______________**

| \| Please rate the following statements based on the observed session:  1 = Strongly Disagree  2 = Disagree  3 = Not sure  4 = Agree  5 = Strongly Agree \| \| --- \| | | | | **Strongly Disagree** | **Disagree** | **Not sure** | | **Agree** | **Strongly Agree** | **Not Applicable** |
| --- | --- | --- | --- | --- | --- | --- | --- | --- | --- | --- | --- |
| **Applicability and Clarity** | | | |  |  |  | |  |  |  |
| 1. Recommendations offered to the case-presentation were delivered simply and clearly | | | |  |  |  | |  |  |  |
| 1. Recommendations offered were applicable and relevant to the case-presentation | | | |  |  |  | |  |  |  |
| 1. Recommendations offered were specific | | | |  |  |  | |  |  |  |
| 1. Recommendations were unambiguous (e.g., not open to interpretation) | | | |  |  |  | |  |  |  |
| 1. Recommendations offered were “actionable” (e.g., within the scope of responsibility of treating clinician) | | | |  |  |  | |  |  |  |
| 1. Potential resource implications of applying the recommendations have been considered | | | |  |  |  | |  |  |  |
| **Content validity** | | | |  |  |  | |  |  |  |
| 1. Specific recommendations offered were consistent with national/international standards of care | | | |  |  |  | |  |  |  |
| 1. Recommendations were timely to current national/international standards of care | | | |  |  |  | |  |  |  |
| 1. References and systematic evidence were cited as evidence for recommendations. | | | |  |  |  | |  |  |  |
| 1. Strengths and limitations of the recommendations are clearly described | | | |  |  |  | |  |  |  |
| **Stakeholder Engagement** | | | |  |  |  | |  |  |  |
| 1. The views and preferences of colleagues and other experts in the panel were sought when recommendations were presented | | | |  |  |  | |  |  |  |
| 12.) Encourages participation and questions from audience after recommendations are presented and discussed (e.g., “Are there any questions from audience?”) | | | |  |  |  | |  |  |  |
| **Documentation and follow-up plan** | | | |  |  |  | |  |  |  |
| 13.) A plan to follow-up and monitoring of the recommendations are shared with the audience/written notes | | | |  |  |  | |  |  |  |
|  | **Very poor** | **Poor** | **Fair** | **Good** | | | **Excellent** | | |  |
| **14.) Rate the overall quality of these recommendations** |  |  |  |  | | |  | | |  |

**Additional comments from observations:**
