# Supplementary material for: A Protocol for a Comprehensive Monitoring and Evaluation Framework With a Compendium of Tools to Assess Quality of Project ECHO (Extension for Community Healthcare Outcomes) Implementation Using Mixed Methods, Developmental Evaluation Design
Source: Front Public Health. 2021 Sep 21;9:714081. doi: 10.3389/fpubh.2021.714081 (PMC8491604; doi:10.3389/fpubh.2021.714081)
Supplement: Supplementary file 1 [file Data_Sheet_1.zip › Appendix 5.docx]

**Appendix 5**

**Routine HIV ECHO program and iECHO Review Data Collection Sheet**

**External objective evaluator will analyze data dump from iECHO sites and tracker tool**

**Objective for this analysis is to ascertain information collected currently, determine content, audience, need, reason, and frequency of use of this data. This tool will help synthesize quarterly participant barrier and satisfaction with ECHO.**

1. **Number of HIV ECHO sessions conducted (Trends? Patterns of users – attended once, vs all ):**
2. **Time period of analysis:**
3. **Number of topics covered:**
4. **Obtain list of all topics:**
5. **Number of participants attending each session:**
6. **Number of core faculty:**
7. **Number of guest/expert faculty:**
8. **Number of geographic locations of participation**
9. **Number and type of specialties**
10. **Number of Health center names**
11. **How is this information being shared currently?**
12. **With who is this data being shared? How often?**
13. **What would be useful to share? How often?**
14. **How is the communication via tracker correlating participants and topics on iECHO?**

**Benefits and Barriers Assessment:**

Please rate your level of agreement with the following statements:

|  | Strongly Disagree | Disagree | Neutral | Agree | Strongly Agree |
| --- | --- | --- | --- | --- | --- |
| The information presented has been relevant to my work | 1 | 2 | 3 | 4 | 5 |
| The information presented has increased my knowledge about HIV care and treatment | 1 | 2 | 3 | 4 | 5 |
| The information presented has increased my ability to better manage the care of my patients with HIV | 1 | 2 | 3 | 4 | 5 |
| The information presented has led me to make changes in my practice | 1 | 2 | 3 | 4 | 5 |
| Clinic duties do not prevent me from attending HIV ECHO | 1 | 2 | 3 | 4 | 5 |
| I get reminders to attend HIV ECHO | 1 | 2 | 3 | 4 | 5 |
| I am available to attend at the time of the HIV ECHO clinic | 1 | 2 | 3 | 4 | 5 |
| I feel comfortable speaking during an HIV ECHO session | 1 | 2 | 3 | 4 | 5 |
| I feel comfortable asking questions during and HIV ECHO session | 1 | 2 | 3 | 4 | 5 |
| I feel comfortable presenting a case during HIV ECHO | 1 | 2 | 3 | 4 | 5 |

**Professional Satisfaction Assessment:**

Please rate your level of agreement with the following statements:

|  | Strongly Disagree | Disagree | Neutral | Agree | Strongly Agree |
| --- | --- | --- | --- | --- | --- |
| I feel professionally isolated at work | 1 | 2 | 3 | 4 | 5 |
| I can connect with professional peers easily | 1 | 2 | 3 | 4 | 5 |
| I can easily access clinical specialists when I need professional feedback/assistance | 1 | 2 | 3 | 4 | 5 |
| I have an opportunity to share clinic experience with colleagues on a regular basis | 1 | 2 | 3 | 4 | 5 |
| I feel satisfied with my job | 1 | 2 | 3 | 4 | 5 |
| I am confident that I can improve the overall quality of services at my health facility | 1 | 2 | 3 | 4 | 5 |
