# Supplementary material for: A Protocol for a Comprehensive Monitoring and Evaluation Framework With a Compendium of Tools to Assess Quality of Project ECHO (Extension for Community Healthcare Outcomes) Implementation Using Mixed Methods, Developmental Evaluation Design
Source: Front Public Health. 2021 Sep 21;9:714081. doi: 10.3389/fpubh.2021.714081 (PMC8491604; doi:10.3389/fpubh.2021.714081)
Supplement: Supplementary file 1 [file Data_Sheet_1.zip › Appendix 6.docx]

**Appendix 6: Readiness Assessment Questionnaire**

**Semi-structured Interview to assess HIV ECHO Implementation readiness**

**(To be completed by a new HIV ECHO coordinator who is planning to implement HIV ECHO outside of Dar es salaam)**

**Completed by on / / (Date) State:**

**(Please circle the correct responses)**

1. Are you planning to start a HIV ECHO this year?  **Yes/No/Not sure**
2. Did you engage with other HIV ECHO implementers prior to the launch of your HIV ECHO programme? **Yes/No/Not sure**
3. Who was/were they?
4. How did you communicate with these implementers?
5. Have you identified a target audience for your ECHO sessions? **Yes/No/Not sure**
   1. If yes, are there varying skill sets and competencies within the target audience? **Yes/No/Not sure**
   2. If not, how are you planning to develop the topics for your target audience?
6. Have you developed a curriculum for your programme? **Yes/No/Not sure**
7. If skills of target audience vary, have you developed a separate curriculum for each skill set and competency?
8. Have you identified “core faculty” to facilitate and implement your ECHO sessions? **Yes/No/Not sure**
9. If yes, have you developed a schedule for HIV ECHO sessions? **Yes/No/Not sure**
   1. Case studies **Yes/No/Not sure**
   2. Didactics? **Yes/No/Not sure**
   3. Are the case studies related to the didactics?  **Yes/No/Not sure**

**(Please write the selected number in the boxes next to the questions)**

|  | **Assessment element** | **Strongly Agree**  **1** | **Agree**  **2** | **Neutral**  **3** | **Disagree**  **4** | **Strongly Disagree**  **5** | **Notes** |
| --- | --- | --- | --- | --- | --- | --- | --- |
| 10. | Do you believe that the ECHO model aligns with the broader HIV prevention and care activities of MOH to reach Tanzania HIV epidemic control strategy of 2022? |  |  |  |  |  |  |
| 11. | Do you believe that there may be funds/resources available outside your office to help implement and sustain HIV ECHO? |  |  |  |  |  |  |
| 12. | Do you believe that the ECHO model promotes partnerships for healthcare in Tanzania? |  |  |  |  |  |  |
| 13. | Do you believe that your organization’s approach matches ECHO’s philosophy of democratization of knowledge, bidirectional knowledge exchange, and removal of hierarchical learning? |  |  |  |  |  |  |
| 14. | Do you believe that the ECHO model will be seen as an important and valuable intervention for your program? |  |  |  |  |  |  |
| 15. | Could your organization easily integrate the ECHO model within existing organizational structures, workflows, and systems? |  |  |  |  |  |  |
| 16. | Do you believe that your team members (operations and hub) are aligned with the ECHO learning philosophy? |  |  |  |  |  |  |
| 17. | Do you believe you will be able to recruit experts in the field, who can serve as mentors to others during this intervention? |  |  |  |  |  |  |
| 18. | Do you believe your organization is sufficiently connected to other partners to support complex networks, communications, and relationships required to sustain ECHO implementation? |  |  |  |  |  |  |
| 19. | Is your organization flexible, willing to make mid-course adjustments to ECHO implementation, as new tools, regimens and approaches emerge? |  |  |  |  |  |  |
| 20. | Are you financially able to pilot an ECHO project without external funding? |  |  |  |  |  |  |
| 21. | After reviewing a sample ECHO budget, do you believe your organization is willing and able to support the costs (staffing, financial, infrastructure, and opportunity costs) associated with the ECHO model through either personal, organizational, or external funding? |  |  |  |  |  |  |
| 22. | Have you attended an ECHO immersion-training program conducted by UNM? |  |  |  |  |  |  |
| 23. | Do you believe that the ECHO immersion training helps support training and implementation? |  |  |  |  |  |  |
| 24. | What is your plan to document recommendations provided during HIV ECHO sessions? |  | | | | | |
| 25.  (i)  (ii)  (iii) | Is there a plan that recommendations given at a HIV ECHO session will be followed-up on?  How?  Where will the recommendations be documented?  How often are you planning to follow-up on the recommendations? |  | | | | | |

26. Any other key considerations or concerns that is bothering you as you think about ECHO implementation?

27. What barriers do you anticipate before starting your ECHO sessions?

1. Cost **Yes/No/Not sure**
2. Time spent on planning/coordination/implementation? **Yes/No/Not sure**
3. ECHO equipment **Yes/No/Not sure**
4. Broadband connection **Yes/No/Not sure**
5. IT support and training **Yes/No/Not sure**
6. Available staff **Yes/No/Not sure**

(vii) Training of staff **Yes/No/Not sure**

1. Motivation of participants **Yes/No/Not sure**

(ix) Knowledge need and understanding from participants (Stakeholder meeting) **Yes/No/Not sure**

(x) Interest from participants **Yes/No/Not sure**

28. Do you have plans to routinely monitor or evaluate your HIV ECHO programs once they are implemented? **Yes/No/Not sure**

28. (i) If there are plans, please elaborate:

29. Anything else that is worrying you that has not been mentioned previously?

30. Other comments: Anything that you wish you knew before you committed to initiating HIV ECHO program?

Thank you! (Asante!)
