# Supplementary material for: A Protocol for a Comprehensive Monitoring and Evaluation Framework With a Compendium of Tools to Assess Quality of Project ECHO (Extension for Community Healthcare Outcomes) Implementation Using Mixed Methods, Developmental Evaluation Design
Source: Front Public Health. 2021 Sep 21;9:714081. doi: 10.3389/fpubh.2021.714081 (PMC8491604; doi:10.3389/fpubh.2021.714081)
Supplement: Supplementary file 1 [file Data_Sheet_1.zip › Appendix 7.docx]

**Appendix 7: Key-informant interview Guide**

***Assess feasibility of implementing the evaluation framework with compendium of tools to measure high-quality ECHO implementation routinely***

***Introduction, explanation of the process and consent review:***

As you are aware, we are developing an objective framework to assess and monitor high-quality HIV ECHO implementation. This format will help us better understand your personal opinion of the process and tools we created, give you an opportunity to respond in greater detail, and offer suggestions for improvement. This semi-structured interview may take approximately 90 minutes. All of your responses will be kept confidential. Your participation has minimal risk and shall not affect your employment status, or relationship or collaboration with any of the ECHO programs and partners.

Your participation is completely voluntary. You need not respond to all questions. If you are unable or unwilling to respond to a specific question, please make me aware and we will move on to the next. We may stop the interview at any time should you become uncomfortable. I will be audio recording this interview to ensure that I accurately and completely capture your responses. Once the interview is complete, I will transcribe the recording into a word document, which I will share with you so that you can validate the accuracy. Once the transcript is complete, I will destroy today’s recording. No names or other identifying information will be recorded.

Do you have any questions for me? Are you willing to participate in today’s interview?

Signature:

Name:

Great, if you are ready to ready to begin, the first question is…

**Date of Interview:**

**Role in ECHO implementation (UMB Implementer/ MOH/CDC-Tanzania staff):**

| **Overall framework feedback** | **Questions** | **Responses** |
| --- | --- | --- |
| **Factors to validate high-quality HIV-ECHO implementation** | | |
| 1. **Capacity building and knowledge dissemination** | 1.) Can you share an example of how you think HIV ECHO is building capacity by sharing knowledge on how to diagnose, treat, and manage complex HIV cases?   1. What are some of the ways you promote knowledge dissemination to engage partners? 2. Tell me what you think is the best way to routinely measure and monitor impact of the capacity building activities? 3. In your opinion, which tools from the compendium could be used to measure knowledge dissemination and capacity building? 4. The provider survey included some questions to measure impact. In your opinion, to what extent did the survey measure impact? What would you add to the survey that could measure impact of capacity building activities? What would you remove from the survey? 5. How well do you think the FGD captures the results of probing questions on capacity building activities?      1. Can you share your ideas on how capacity building activities should be monitored and evaluated as the national HIV ECHO expansion evaluation strategy? 2. How often do you think capacity building should be measured? Continued Medical Education (CME) credit related questionnaires are usually administered after ECHO sessions end in other countries. Would you consider the standard 5-10 question CME survey a capacity building measurement [Probe: Every session, Quarterly, Semi-annually, Annually] 3. How often should the survey be administered? And the FGD? 4. Who should lead this effort? MOH? CDC Tanzania? UMB? Other? |  |
| **2.) Engaging partners** | 1. What can you tell me about the partnerships you have built or expanded through HIV ECHO?   [Probe: Who are these partners?]   - 1. Are you satisfied with them?   2. How did you recruit or engage them?  1. What does high-quality partnerships mean to you? 2. In your opinion, how would you measure establishment of such relationships with partners? 3. Can you give me an example of ways you could monitor quality of these partnerships, and who have been involved in these partnerships? 4. Can you share an example of how you think you can sustain these partnerships?   [Probe: Since inception of ECHO, what are some of the ways you have been able to expand partners, and with who?]   1. Can you give an example of how you would measure the impact of partnerships routinely? 2. Who should be responsible for establishing partnerships? And measuring impact of such relationships? 3. The provider survey includes some questions to measure establishing partnerships. To what extent did the survey capture this? 4. How often should quality of partnership interactions partnerships be measured for M&E purposes?   [Probe: Every session, Quarterly, Semi-annually, Annually]   1. What indicators do you think national HIV ECHO expansion evaluation strategy should include to measure impact of partnerships? 2. Who should be responsible for monitoring this routinely? |  |
| **3.) Establishing communities of practice (CoP)** | 1. What can you tell me about the establishment of communities of practice through HIV ECHO? ***By community of practice, I mean a group of people who share a concern or a passion for something, so in this situation, HIV/TB, share knowledge and experience and learn how to do it better as they interact regularly through these HIV ECHO Sessions*** 2. What would such a communities of practice look like?   Probe: Membership? Activities?  Probe: Do you consider yourselves building such communities of practice? Do you consider the current HIV ECHO a CoP?   - 1. Are you satisfied with this CoP? Why? Why not?   2. If not, what could you do differently?  1. What you think is the best way to measure and monitor ECHO communities of practice? In your opinion, how would you best monitor and sustain communities of practice? 2. How would you measure the impact of communities of practice? 3. In your opinion, which tools from the compendium could be used to measure community of practice and engage partners? 4. The provider survey and focus group guide included some questions aimed at measuring the impact of communities of practice. To what extent did the survey capture this? FGD? 5. How often do you think the impact of communities of practice should be measured? [Probe: Every session, Quarterly, Semi-annually, Annually]   29.) Usually an effective ECHO program builds CoP. Who, in your opinion should be responsible for building communities of practice as ECHO is expanded?  30.) Do you think national HIV ECHO evaluation strategy should include indicators to measure the impact of Communities of Practice?  31.) Who should lead the effort to measure impact of CoP routinely? |  |
| **4.) Administration and Resources** | 1. From your experience, about how much time, resources, and people are needed to implement a high-quality HIV ECHO? 2. How much time is spent coordinating individual sessions? 3. How much time is spent developing course content? 4. In your experience, who is coordinating/leading the administration and logistics of the ECHO sessions? 5. How much time do you think the experts (case presenters and didactic presenters) are spending on preparing for the sessions? 6. How much time is time spent recruiting faculty to present?      1. Can you give an example of when you have observed or heard about participants integrating lessons learned into practice? 2. Approximately how much time is spent on administrative aspects of ECHO activities?   40.) How much time is spent on routine monitoring and evaluation activities?  41.) How much time is spent on debriefing after ECHO sessions?  42.) What resources would you wish you had for high-quality ECHO implementation?  43.) How important is it to monitor administrative and financial resources routinely?  44.) Who should lead this effort for HIV ECHO management and administration of resources? |  |
| **5.) Measuring public health impact** | 45.) Give me an example of how you could measure the overall public health impact of ECHO? (E.g., measure if the sessions are improving patient care and outcomes)  46.) How can some of these indicators be collected routinely?  47.) What resources would we need for that?  48.) How often should measuring public health impact be monitored in your opinion?  [Probe: Every session, Quarterly, Semi-annually, Annually]  49.) Who should lead this effort for measuring public health impact routinely? |  |
| **6.) Course Content** | 50.) Now that you have reviewed the results of the objective review of the course content, what are some of the things that resonated with you from that objective exercise?  51.) What can be done to improve that process? What would you do differently?  52.) What are your thoughts about next steps needed to improve and maintain the quality of your course content?  53.) Does it make sense to do this objective review routinely?  54.) In your opinion, how often should the course content evaluated by third party, objective reviewers? [Probe: Semi-annually, Annually]  55.) How much do you think the medical community values continuing medical education (CME) credits for participants or certificates of appreciation to experts or case presenters?  56.) Who would most value receiving official certificates or CMEs?  57.) To what extent is such a CME system already established or how feasible would it be to establish one for this group?  58.) What resources would need to be put in place for this system?  59.) Who should lead this effort to manage quality and delivery of high-quality ECHO sessions? |  |
| **7.) IT and logistical support** | 60.) How would you rate on a 1 to 10 scale (1 being least and 10 being the best), the IT and logistical support from UMB?  61.) What kinds of IT or logistical support have you sought assistance for?  62.) How satisfied were you with the help you received?  63.) What recommendations can you give for it to be improved?  64.) What would be your advice for new HIV ECHO sites beginning implementation with regards to IT and logistic challenges?  65.) How can they best remedy this challenges?  66.) Are you familiar with the iECHO software?  67.) Can you share your thoughts on how iECHO impacts your work or ECHO experience? |  |
| **8.) Conclusion** | Anything else you would like to comment on about HIV ECHO that I have not asked about? |  |
| **STOP**  **unless interviewing an implementer** | | |
| **Preparation for HIV ECHO sessions (Ask implementers)** | | |
| Pre-session preparation Activities | Think back to a HIV ECHO session that you were particularly satisfied with:     1. Tell me about the process of how you recruited the presenter? 2. What information is shared with the case presenter to ensure integrity and relevance of the sessions? 3. How long before a HIV ECHO session do you identify a case presenter to present a case from their practice?   D. What information is shared with the didactic presenter to ensure integrity and relevance of the topics between sessions?  E. Does someone from your team review the presentations prior to the session to ensure quality? That the content is accurate? Current and consistent with national guidelines? Free from commercial bias or other conflict of interest?  F. Does someone from your team review the case presentations prior to the session to ensure quality? That the content is sufficient for others learning?  G. In preparation for the session, does the course facilitator do any “prep work”? Reviewing guidelines? Conduct literature reviews? Become familiar with the didactic presentation? The case presentation?  H. In preparation for the session, do the faculty mentors (experts) do any “prep work”? Reviewing guidelines? Conduct literature reviews? Become familiar with the didactic presentation? The case presentation?   1. What are the most difficult parts of making all ECHO sessions go this well? |  |
|  | J. Thoughts on how iECHO or a system to monitor outcomes could be implemented in India? |  |
| **Recommendations and Follow-up from ECHO sessions** | | |
| After ECHO sessions | K. What kinds of processes are in place for systematically following up with recommendations?  L. With respect to the individual case recommendations provided by faculty mentors, are there processes in place to systematically document the clinical recommendations? Are these recommendations added to the patient records in any way? If so, where? Are the recommendations only shared with the case presenter, or also with the entire community of practice?  M. Can you think of an example of changes practice or policy following an ECHO session or recommendation? Do you think individual recommendations are applied to other patients or to future patients? |  |
|  | N. In your opinion, is there a way to ensure that individual recommendations given at a HIV ECHO clinic are acted upon?  O. How often does your team follow-up on a previously presented case? If not happening now, do you think monitoring individual cases presented is important? If so, is it feasible? How often would case follow-up be reasonable?  P. Who should be responsible for following up and documenting after ECHO sessions?  Q. What kinds of quality improvement processes do you have in place for any of the activities we have just discussed?  R. Are there any grand rounds of presentation of problem cases or cohort review style in ECHO sessions?) Problems encountered and how they are being solved? |  |
| **Conclusion** | 68.) Anything else you would like to comment on about HIV ECHO that I have not asked about? |  |
| **Design of the evaluation framework** | 69.) Now that you have thought about the different concepts that were identified in the environmental scan workshop, what feedback do you have on the evaluation framework, compendium of tools, and some of the preliminary results, what is your impression of the evaluation framework (Appendix 1)? [Show Appendix 1]    a.) What’s your overall impression?  b.) What changes would you recommend? | |

**Summary Checklist (so to summarize what I heard from you are the following):**

| **Factors to measure high-quality ECHO implementation** | **Tool(s)** | **Frequency of assessment** | **Who should be responsible?** |
| --- | --- | --- | --- |
| Capacity building and knowledge dissemination | - SOAR Appreciative inquiry process to check-in six-month post HIV ECHO implementation - Provider survey - FGD |  |  |
| Engaging partners | - Provider survey - FGD - iECHO analysis |  |  |
| Expanding communities of practice | - SOAR process to check-in six-month post HIV ECHO implementation - Readiness assessment |  |  |
| Administration and resources | - Provider survey - SOAR Appreciative inquiry process to check-in six-month post HIV ECHO implementation - Readiness Assessment |  |  |
| Political will and support | - Provider survey - Readiness assessment |  |  |
| Content Review | Objective content review tools:   - Facilitator session assessment - Content assessment - Recommendation review |  |  |
| Measuring public health impact | - Provider survey - Readiness assessment   iECHO analysis |  |  |
| Documentation of long-term outcomes | - FGD - Readiness assessment - Objective content review |  |  |

**70. What do you think should be the priority for the next steps?**

**Other comments?**
