# Supplementary material for: A Protocol for a Comprehensive Monitoring and Evaluation Framework With a Compendium of Tools to Assess Quality of Project ECHO (Extension for Community Healthcare Outcomes) Implementation Using Mixed Methods, Developmental Evaluation Design
Source: Front Public Health. 2021 Sep 21;9:714081. doi: 10.3389/fpubh.2021.714081 (PMC8491604; doi:10.3389/fpubh.2021.714081)
Supplement: Supplementary file 1 [file Data_Sheet_1.zip › Appendix 8.docx]

**Appendix 8: HIV ECHO Key Informant Interview Consent Form**

**Introduction**

The HIV ECHO offers an opportunity to build bridges between MOH, UMB, CDC Tanzania, and local community health partners in an ‘all teach, all learn’ interactive format. To understand the impact of HIV ECHO, University of Illinois in Chicago in collaboration with Centers for Disease Control and Prevention (CDC) are developing an evaluation framework and pilot testing data collection tools. You are being invited to participate in this key informant interview because you have been identified as a potential influencer, and your opinions will add value to our understanding the various perspectives of HIV ECHO implementation in Tanzania.

**What will happen if I decide to participate?**

If you agree to participate, you shall be interviewed by a study investigator in a quiet and confidential setting. This interview may last up to 90 minutes. Interviews will be digitally recorded and transcribed, no personally identifiable information will be collected.

**What are the risks or side effects of being interviewed?**

There are minimal risks of discomfort when answering questions. Discussions about sensitive personal information will be discouraged but open and honest opinions shall be welcomed.

**What are the benefits to being interviewed?**

By participating in an interview, you will be helping us better understanding the elements needed for high-quality HIV ECHO implementation. The outcome of our work will produce a systematic and comprehensive process for monitoring and evaluating ECHO programmes.

**How will my information be kept confidential?**

Digital recordings and transcriptions will remain in a locked, secure location within the CDC office during our analysis phase. We will destroy these records and recordings at the completion of the study.

**Can I stop being in the interview once I begin?**

Your participation is completely voluntary. You have the right to choose not to participate or to withdraw your participation at any point during this interview. Non-participation or withdrawal will NOT affect your employment or participation in future HIV ECHO activities.

**Whom can I call with questions or complaints about this interview?**

If you have any questions, concerns or complaints at any time, please contact the study Principle Investigator:

(PI Information)

**CONSENT**

I agree to participate in this key informant interview. I acknowledge the potential risk and benefits of participating, and understand that I may withdraw at any time.

_____________________________________ ___________________

Participant, Name [Print], Signature Date

_____________________________________________________________

Investigator, Name [Print], Signature Date

_____________________________________________________________

Witness, Name [Print], Signature Date
