# Supplementary material for: A Protocol for a Comprehensive Monitoring and Evaluation Framework With a Compendium of Tools to Assess Quality of Project ECHO (Extension for Community Healthcare Outcomes) Implementation Using Mixed Methods, Developmental Evaluation Design
Source: Front Public Health. 2021 Sep 21;9:714081. doi: 10.3389/fpubh.2021.714081 (PMC8491604; doi:10.3389/fpubh.2021.714081)
Supplement: Supplementary file 1 [file Data_Sheet_1.zip › Appendix 9.docx]

**Appendix 9**

**Confidentiality Agreement**

**Statement of Intent to Maintain Confidentiality**

**Project Title*:*** **Developing a comprehensive monitoring and evaluation framework with a compendium of tools for a high-quality HIV Extension for Community Health Outcomes (HIV ECHO) implementation – Tanzania**

As a member of this evaluation team, I understand that I may have access to confidential information about evaluation sites and participants. By signing this statement, I am indicating my understanding of my responsibilities to maintain confidentiality and agree to the following:

- I understand that all information about evaluation sites or participants obtained or accessed by me in the course of my work is confidential. I agree not to divulge or otherwise make known to unauthorized persons any of this information, unless specifically authorized to do so by approved protocol or by the local principal investigator acting in response to applicable law or court order, or public health or clinical need.
- I understand that names and any other identifying information about the evaluation sites and participants are completely confidential and will not be shared outside the study team.
- I agree not to divulge, publish, or otherwise make known to unauthorized persons or to the public any information obtained in the course of this evaluation that could identify the persons who participated in the study.
- I agree to notify the local principal investigator immediately should I become aware of an actual breach of confidentiality or a situation which could potentially result in a breach, whether this be on my part or on the part of another person.

______________________________ _________________ ________________________

Signature Date Printed Name
